# New Zinc(II) Coordination Compound with 1,10-Phenanthroline and Maleate: Comprehensive Structural Analysis, Periodic-DFT Calculations, and Evaluation of Biological Potential

**DOI:** 10.1021/acsomega.5c09973

**Published:** 2026-01-22

**Authors:** João G. de Oliveira Neto, Jailton R. Viana, Anna R.P. Valerio, Otávio C. da Silva Neto, Luiz F. L. da Silva, Alejandro P. Ayala, Eliana B. Souto, Adenilson O. dos Santos, Rossano Lang

**Affiliations:** a Center for Social Sciences, Health and Technology, Federal University of Maranhão (UFMA), Imperatriz, MA 65900-410, Brazil; b Institute of Science and Technology, Federal University of São Paulo (UNIFESP), São José dos Campos, SP 12231-280, Brazil; c Higher Education Institute of Southern Maranhão, Higher Education Unit of Southern Maranhão (UNISULMA), Imperatriz, MA 65907-070, Brazil; d Criminalistics Institute, Scientific Police of Para, Marabá, PA 68507-000, Brazil; e Department of Physics, Federal University of Ceara (UFC), Fortaleza, CE 65455-900, Brazil; f UCD School of Chemical and Bioprocess Engineering, 8797 University College Dublin, Belfield, Dublin 4 D04 V1W8, Ireland

## Abstract

The escalating crisis of bacterial resistance necessitates the development of novel antimicrobial agents. Herein, we report the synthesis and comprehensive characterization of a new zinc­(II) coordination compound, [Zn­(phen)­(maleate)­(H_2_O)]·H_2_O (phen = 1,10-phenanthroline). Single-crystal X-ray diffraction revealed a distorted square pyramidal geometry around the Zn­(II) center, forming a supramolecular framework (triclinic, P1̅ ) stabilized by hydrogen bonding (H···O/O···H: 30.6%) and π–π stacking interactions (C···C: 9.0%), as quantified by Hirshfeld surface analysis. Periodic density functional theory (DFT) calculations confirmed a direct energy gap of 3.45 eV and thermodynamic stability under ambient conditions. Vibrational spectroscopy (infrared and Raman) combined with DFT calculations provided suitable mode assignments. The compound exhibited selective antibacterial activity against Gram-positive *Streptococcus mutans* (MIC = 1000 μg/mL) with no activity against Gram-negative *Escherichia coli*. Systematic control experiments confirmed that antibacterial activity originates from the intact coordination complex rather than individual components. *In silico* pharmacokinetics predictions indicated favorable gastrointestinal absorption, full compliance with drug-likeness rules (Lipinski, Ghose, Veber, Egan, Muegge), and no cytochrome P450 inhibition. Molecular docking studies revealed specific binding to a *S. mutans* enzyme (ΔG = −7.4 kJ/mol), suggesting enzyme inhibition as the primary mechanism. This work establishes a multidisciplinary framework for rational Zn-coordination compounds design while highlighting critical needs for toxicological validation and structural optimization to enhance potency and broaden antimicrobial spectrum.

## Graphical abstract

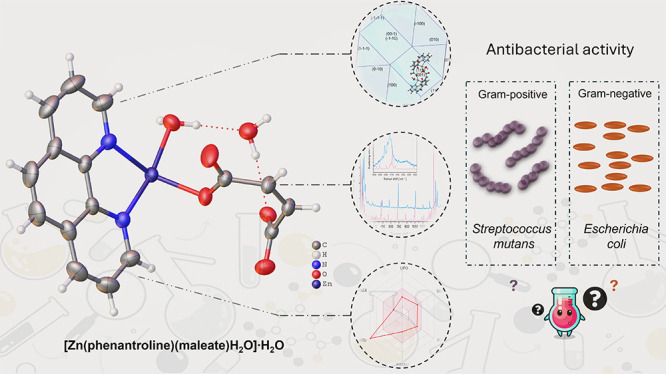

## 1 Introduction

The escalating crisis of bacterial resistance represents one of the most pressing global health challenges, severely undermining the efficacy of conventional antibiotics and driving an urgent need for novel antimicrobial agents with distinct mechanisms of action.^1,2^ In this context, coordination compounds have gained prominence as promising alternatives, not only due to their remarkable structural diversity, but also for their ability to fine-tune physicochemical and biological properties through the suitable selection of metal ions and organic ligands.^3,4^ This versatility enables the rational design of complexes with potential antimicrobial activity, offering innovative pathways to address resistant infections.^5^ This work directly addresses this critical need via the design and comprehensive characterization of a novel zinc­(II) coordination compound, [Zn­(phenanthroline)­(maleate)­(H_2_O)]·H_2_O, which has shown significant and selective antibacterial activity.

Zinc­(II) complexes are particularly attractive for biomedical applications due to their low toxicity, redox stability, and structural versatility.^6,7^ Critically, zinc-based compounds have shown compelling antibacterial properties against a range of pathogens, including resistant strains, making them excellent candidates for developing new anti-infective therapies.^8,9^ The ability to incorporate bioactive organic ligands further enhances their potential to interact with biological targets and disrupt essential bacterial processes.^10^

The strategic selection of 1,10-phenanthroline (phen) and maleate as ligands is founded on principles of structural and functional complementarity. Phen provides a rigid, aromatic structure with excellent chelating ability through its nitrogen donors, while also enabling stabilizing π-π stacking interactions that direct supramolecular assembly.^11^ The maleate anion contributes with flexible coordination modes via its carboxylate groups and acts as an effective hydrogen-bonding bridge, facilitating the formation of extended lattice.^12^ This specific combination was chosen to exploit the synergy between the DNA-intercalating potential of phen and the maleate’s role in enhancing biological activity and supramolecular organization, key factors influencing bioavailability and antibacterial efficacy.^13,14^

Although the Cambridge Structural Database contains numerous Zn-phen structures,^15^ detailed investigations of systems incorporating dicarboxylates like maleate, particularly with coupled experimental and theoretical analyses of their supramolecular chemistry and antibacterial properties, remain scarce.^16,17^ Most existing reports focus primarily on structural elucidation, overlooking deeper electronic structure–property relationships and quantitative analysis of the noncovalent interactions governing crystal packing and stability.^18,19^ The integration of periodic density functional theory (DFT) calculations, Hirshfeld surface analysis, and void mapping with traditional characterization methods provides a powerful framework to bridge this gap, enabling a more predictive approach to materials design.^20–23^

Herein, we report the synthesis, structural characterization, and biological evaluation of the new compound [Zn­(phen)­(maleate)­(H_2_O)]·H_2_O. Single-crystal X-ray diffraction (XRD) reveals a distorted square pyramidal zinc center within a supramolecular framework stabilized by hydrogen bonding and π-π interactions. A suite of computational techniques including periodic DFT, Hirshfeld surface, and band structure analyses provides deep insight into the electronic structure, thermodynamic properties, and the precise nature of the intermolecular interactions that underpin the crystalline framework. The antibacterial activity Zn­(II) coordination compound was evaluated against Gram-positive *Streptococcus mutans* and Gram-negative *Escherichia coli*, demonstrating selective inhibition. Furthermore, *in silico* absorption, distribution, metabolism, and excretion (ADME) predictions assessed its potential as a lead compound. Stability experiments involving pH variation and molecular anchoring were performed to assess the stability of the compound under diverse physicochemical conditions, as well as to elucidate potential mechanisms of interaction with biomolecular targets.

This study exemplifies a multidisciplinary strategy that integrates coordination chemistry, solid-state physics, computational modeling, and microbiology. It not only presents a new antibacterial candidate but also establishes a comprehensive structure–activity relationship, providing valuable insights and a validated approach for the rational design of next-generation metallodrugs to combat the growing threat of bacterial resistance.

## 2 Experimental and Theoretical Methodology

### 2.1 Synthesis Process

The coordination compound [Zn­(phen)­(maleate)­(H_2_O)]·H_2_O was synthesized by the slow solvent evaporation method, as depicted in Figure 1. The synthesis was adapted from Simplicio et al.,^24^ where the reagents 1,10-phenanthroline monohydrate (C_12_H_8_N_2_·H_2_O, Synth ≥ 99%), maleic acid (C_4_H_4_O_4_, Sigma-Aldrich ≥ 99%), and zinc chloride (ZnCl_2_, Sigma-Aldrich ≥ 98%) were weighed in molar ratios of 2:3:2 (0.9911 g: 0.8702 g: 0.6815 g), respectively. Initially, C_4_H_4_O_4_ and ZnCl_2_ were dissolved in 25 mL of deionized water under magnetic stirring at 360 RPM for 60 min. In parallel, a solution of C_12_H_8_N_2_·H_2_O was prepared in 15 mL of ethanol (CH_3_CH_2_OH, Sigma-Aldrich ≥ 99.5%) under identical stirring conditions. After homogenization, the C_12_H_8_N_2_·H_2_O solution was slowly added dropwise to the C_4_H_4_O_4_–ZnCl_2_ mixture, followed by an additional stirring period of 30 min. Subsequently, the pH of the solution (initially ≈3.7) was adjusted to ≈7.0 using a 0.1 mol/L sodium hydroxide (NaOH, Sigma-Aldrich ≥ 98%) solution. The final solution was stirred for 120 min and filtered using microporous cellulosic filter paper (25 μm).

**Figure 1:**
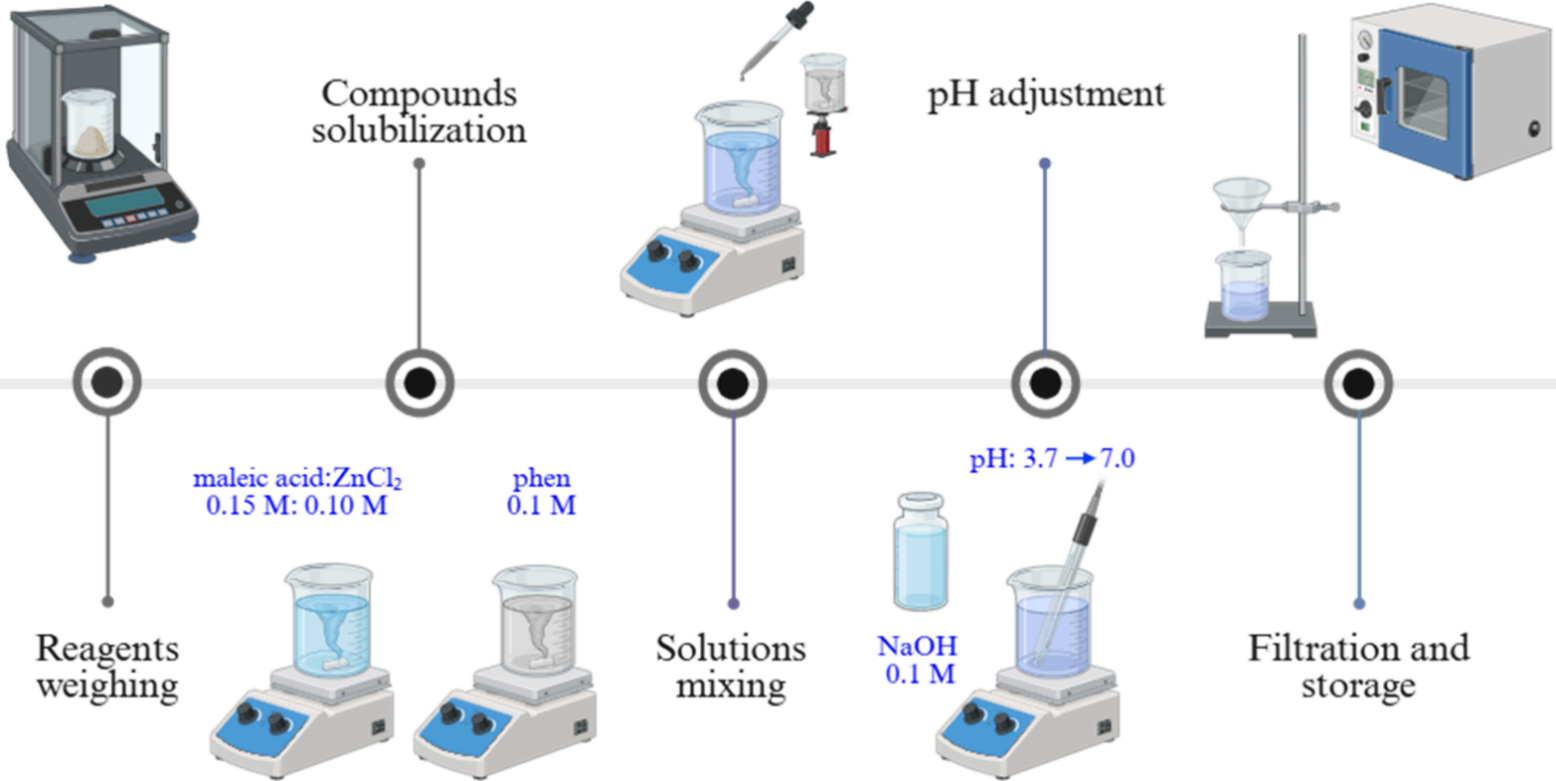
Step-by-step procedure for the synthesis of coordination compound [Zn­(phen)­(maleate)­(H_2_O)]·H_2_O.

The filtrate was covered with plastic film perforated with 15 small holes to allow for controlled solvent evaporation and was subsequently stored in an oven at 308 K (35 °C). After a period of approximately 7 days, opaque white spherical crystals suitable for single-crystal XRD analysis were obtained. The solid product was isolated, washed with acetone (Sigma-Aldrich ≥ 99.5%), and dried under ambient conditions. The yield of the final product was 77%. Elemental analysis was performed to confirm the composition of the obtained crystals and purity. The calculated values were: C - 48.7%, H - 3.6%, N - 7.1%, O - 24.3%, and Zn - 16.3%. The experimentally found values were: C - 48.3%, H - 3.7%; N - 7.0%, O - 24.5%, and Zn - 16.5%. The excellent agreement between calculated and found values confirms the chemical composition of the coordination compound and its high purity.

### 2.2 Structure Determination via Single-Crystal XRD and Analysis of Phase Purity

The structure of the coordination compound [Zn­(phen)­(maleate)­(H_2_O)]·H_2_O was elucidated through single-crystal XRD analysis, performed using a D8 Venture diffractometer (Bruker) equipped with a Photon II CPAD detector and an Incoatec IμS 3.0 Mo Kα microfocus source (λ = 0.71073 Å). A suitable crystal measuring 0.380 × 0.317 × 0.276 mm^3^ was selected for data acquisition and maintained at a constant temperature of 301 K. Initial cell parameters and structural data were obtained using APEX4 software.^25^ Data integration and refinement of the unit cell were carried out with the SAINT software suite,^26^ and absorption effects were corrected via the multiscan method implemented in SADABS.^27^ Structure solving was achieved through intrinsic phasing using ShelXT from the SHELX package,^28^ operated within the Olex2 graphical interface,^29^ enabling the identification of most non-hydrogen atoms. Subsequent refinement cycles employed full-matrix least-squares methods on F^2^ using ShelXL,^30^ with non-hydrogen atoms refined anisotropically. Hydrogen atoms were placed according to idealized geometries and treated using the riding model. Crystallographic information file (CIF) and visual representations were prepared using MERCURY software.^31^ The finalized CIF was submitted to the Cambridge Crystallographic Data Centre (CCDC) under deposition number 2490001. Structural data and geometric parameters are freely accessible at https://www.ccdc.cam.ac.uk/structures/.

The phase purity of the synthesized [Zn­(phen)­(maleate)­(H_2_O)]·H_2_O was confirmed by powder X-ray diffraction (PXRD) using a PANanalytical diffractometer (Empyrean model) with Cu–K_α1_ radiation (λ = 1.5418 Å) operating at 40 kV/40 mA. Data were collected in the 2θ range of 5–50° with a step size of 0.02° and counting time of 2 s per step. In addition, the PXRD pattern was refined by the Rietveld method^32^ using the structural parameters determined by single-crystal XRD with the aid of the GSAS-EXPGUI software.^33^

### 2.3 Thermal Analysis

Thermal stability was investigated by differential scanning calorimetry (DSC) using a DSC-60 thermal analyzer (Shimadzu). Approximately 2 mg of powdered sample was heated from 300 to 700 K under nitrogen atmosphere (100 mL/min) at a heating rate of 10 K/min.

### 2.4 Crystal Voids and Hirshfeld Surfaces

Void regions within the crystal unit cell were identified using electronic density isosurfaces set at 0.002 atomic units, following the approach proposed by Bader et al.^34^ This mapping was generated with the CrystalExplorer software.^35^

Hirshfeld surfaces and two-dimensional (2D) fingerprint plots were also generated using the CrystalExplorer software to further investigate intermolecular interactions among the chemical species in the crystal. The Hirshfeld surfaces were mapped according to the normalized distance (*d*_norm_), which is calculated based on the proximity of each surface point to the nearest internal (*d*_i_) and external (*d*_e_) atoms, incorporating their respective van der Waals radii (*r*_vdW_). Furthermore, the asymmetric unit shape index and surface curvedness were also calculated to support a topological assessment of intermolecular contacts. The 2D fingerprint plots were expressed as a function of *d*_e_ and *d*_i_, capturing the full intermolecular interactions and enabling quantification of specific contact types.^36,37^

### 2.5 Fourier transform infrared (FT-IR) and Raman Spectroscopy

The FT-IR spectrum was obtained using a Bruker Vertex 70 V spectrometer employing the potassium bromide (KBr) pellet method. The samples were blended with approximately 2% anhydrous KBr (purity 98%, Sigma-Aldrich) and subsequently compressed into pellets. Data acquisition was carried out over the spectral window of 4000–400 cm^–1^, with a resolution of 4 cm^–1^ and 32 scans.

The Raman spectroscopy analysis was performed from a nonoriented crystal using a high-resolution triple-stage spectrometer (Princeton Trivista 557) configured in subtractive mode and equipped with a thermoelectrically cooled charge-coupled device (CCD) detector (Horiba). A helium–neon laser providing excitation at 632.8 nm with an output power of ≈165 mW was used as the radiation source. Spectra were acquired with a spectral resolution of 2 cm^–1^, 180^–s^ integration time, and 3 cumulative scans.

### 2.6 Periodic-DFT Calculations

The electronic, optical, vibrational, and thermodynamic properties of [Zn­(phen)­(maleate)­(H_2_O)]·H_2_O were investigated using first-principles calculations based on DFT, as implemented in the Cambridge Serial Total Energy Package (CASTEP).^38^ This software utilizes plane-wave basis sets and pseudopotentials for modeling materials.^39^ Norm-conserving pseudopotentials were employed to treat electron exchange and correlation effects, and the Generalized Gradient Approximation (GGA) with the Perdew–Burke–Ernzerhof (PBE) functional was adopted.^40^ The Brillouin zone was sampled using a 2 × 2× 1 Monkhorst–Pack grid^41^ for the triclinic crystal structure ( $P\mathrm{1̅}\left(C_{i}^{1}\right)$ -space group), which contains a total of 78 atoms. Geometry optimization was performed using the Broyden–Fletcher–Goldfarb–Shanno (BFGS) algorithm,^42^ with convergence criteria set as follows: a maximum force of 0.03 eV/Å, a maximum displacement of 0.001 Å, a maximum energy change of 1.0 × 10^–5^ eV/atom, and a maximum stress of 0.1 GPa. The electronic wave function was propagated along the high-symmetry path in the Brillouin zone of the triclinic phase, following the sequence of points: G­(0.000, 0.000, 0.000); F­(0.000, 0.500, 0.000); Q­(0.000, 0.500, 0.500); Z­(0.000, 0.000, 0.500); G­(0.000, 0.000, 0.000).

### 2.7 Microbiological Experiments

The bactericidal assays were initiated using a 20 mg/mL solution of the [Zn­(phen)­(maleate)­(H_2_O)]·H_2_O powdered crystal in dimethyl sulfoxide (DMSO – Sigma-Aldrich, ≥ 99.9%). Bacterial strains, namely, *Streptococcus mutans* (Gram-positive) and *Escherichia coli* (Gram-negative), acquired from American Type Culture Collection (ATCC), were selected for evaluation. The inoculum was prepared according to the M7 standard (Methods for Dilution Antimicrobial Susceptibility Tests for Bacteria That Grow Aerobically)^43^ to achieve a density equivalent to a 0.5 McFarland standard, corresponding to approximately 1 × 10^8^ CFU/mL, as verified by measuring absorbance between 0.08 and 0.10 at 630 nm using a microplate spectrophotometer (LMR-96 Loccus). Further dilutions were made to adjust the final inoculum to 1 × 10^6^ CFU/mL for the microdilution assay. The broth microdilution was performed in a sterile 96-well polystyrene plate. Initially, 100 μL of Mueller Hinton broth was added to all wells except the first row, where a [Zn­(phen)­(maleate)­(H_2_O)]·H_2_O/DMSO solution (20 mg/mL) was diluted in Mueller Hinton (Sigma-Aldrich) broth to an initial concentration of 1000 μg/mL. Then, 200 μL of this solution was added in triplicate to the first wells, followed by a serial 2-fold dilution, yielding concentrations ranging from 1000 to 7.81 μg/mL. The plate was mixed for 5 min using a microplate shaker and incubated at 308 K for 24 h. For comparative purposes, similar assays were conducted using the standard control Gentamicin (Sigma-Aldrich). To determine the minimum inhibitory concentration (MIC), 0.02% resazurin (Sigma-Aldrich) solution in sterile water was used. After incubation, 20 μL of the resazurin solution was added to each well and incubated for 2–4 h at 308 K, after which the MIC was assessed visually. The MIC was defined as the lowest concentration of the compound that completely inhibited bacterial growth.

To ensure the specificity of the antibacterial activity, appropriate controls were included in the assay. Individual components: ZnCl_2_ (at concentrations equivalent to the Zn^2+^ content in the complex), free phen, and maleic acid (maleate) were tested under identical conditions. Additionally, DMSO solvent controls were performed at the highest concentration present in the assay wells (5% v/v in the first dilution wells, decreasing to 0.08% v/v in the final dilution). The final DMSO concentration in all test wells ranged from 5% to 0.08% v/v across the serial dilutions, concentrations well below the threshold known to affect bacterial growth (≤10% v/v). Visual assessment of bacterial growth was performed by observing color change of resazurin from blue (no growth) to pink (viable bacteria).

### 2.8 *In Silico* Pharmacokinetics

The pharmacokinetic parameters of [Zn­(phen)­(maleate)­(H_2_O)]·H_2_O were evaluated *in silico* using the SwissADME platform.^44^ This tool was utilized to estimate key ADME descriptors, supporting the interpretation of experimental data. Furthermore, the BOILED-Egg model was applied to predict gastrointestinal (GI) absorption and brain penetration, while the radar plot visualization was generated to provide a comprehensive overview of drug-likeness and physicochemical characteristics.

### 2.9 Stability Studies in Different pH Media

The chemical stability of [Zn­(phen)­(maleate)­(H_2_O)]·H_2_O was investigated at different pH values to simulate various biological environments. The compound was dissolved in acidic (pH 3.7), neutral (pH 7.0), and basic (pH 10.5) media. Aliquots were collected at 0, 12, and 24 h and analyzed using a Thermo Evolution 220 UV–vis-NIR double-beam spectrophotometer equipped with a deuterium lamp.

### 2.10 Molecular Docking

Molecular docking simulations were carried out using the AutoDock 4.2 software^45^ to investigate the binding affinity of the compound with DNA (PDB ID: 1BNA) and the cocrystallized enzyme present in *Streptococcus mutans* (PDB ID: 9CY9), whose crystal structures were retrieved from the Protein Data Bank (PDB). Prior to docking, the receptor files were prepared by removing the free water molecules and cofactors, retaining only the structural framework of each receptor. The docking grid was configured to encompass the relevant interaction regions within each macromolecule, and intermolecular interactions were assessed to determine the binding affinity of the compound with specific residues of the receptors. The resulting simulations were analyzed and visualized using Discovery Studio software.^45^

## 3 Results and Discussion

### 3.1 Initial Physical Characterization, Structure, and Thermal Behavior

The new Zn­(II) coordination compound containing the ligands phen, maleate, and H_2_O was successfully synthesized via the slow solvent evaporation method, as described in Equation 1. The synthesis was carried out in a hydroethanolic medium, which facilitated the solubilization of the starting materials and promoted the formation of the coordination compound. Although Zn^2+^ ions were introduced into the coordination sphere through the zinc chloride (ZnCl_2_) salt, the chloride anions (Cl^–^) do not participate in the formation of the final solid product. Instead, they remain in solution, reacting with the Na^+^ ions, originating from the pH adjustment with NaOH in aqueous medium, to form NaCl subproduct. Furthermore, maleic acid (C_4_H_4_O_4_) undergoes deprotonation, converting into its corresponding anionic form, maleate (C_4_H_2_O_4_^2–^). This transformation enables the ligand to act as a chelating, coordinating to the metal center through covalent bonding via the oxygen atoms of the carboxylic groups.

$${\mathrm{ZnCl}}_{2}\left(s\right)+C_{4}H_{4}O_{4}\left(s\right)+C_{12}H_{8}N_{2}\cdot H_{2}O\left(s\right)+2\mathrm{NaOH}\left(\mathrm{aq}\right)\overset{H_{2}O/C_{2}H_{5}OH}{\to }\left[\mathrm{Zn}\left(\mathrm{phen}\right)\left(\mathrm{maleate}\right)\left(H_{2}O\right)\right]\cdot H_{2}O\left(s\right)+2\mathrm{NaCl}\left(\mathrm{aq}\right)+H_{2}O\left(l\right)$$ 1

During the coordination process, in addition to the maleate ligand, Zn^2+^ ions coordinate with two nitrogen atoms from phen and one molecule of H_2_O. Furthermore, a second H_2_O molecule is incorporated into the structure as free water, present in the crystal lattice. This leads to the formation of the coordination compound [Zn­(phen)­(maleate)­(H_2_O)]·H_2_O as a crystalline solid. It is important to highlight the presence of ethanol (C_2_H_5_OH) in the reaction medium, which plays a key role in product crystallization by reducing the solvent polarity and promoting selective nucleation of the compound. Although C_2_H_5_OH is involved during the synthesis, it does not participate directly in the chemical reaction, meaning that it undergoes no transformation and is not incorporated into the final product. Conversely, H_2_O functions both as a component of the reaction medium and as a ligand, owing to its excess.

The [Zn­(phen)­(maleate)­(H_2_O)]·H_2_O crystallized in the form of opaque white spherical crystals, characteristic of polycrystalline systems, as shown in Figure S1. This morphology is commonly associated with radial nucleation and growth processes, typical of spherulite-type structures.^46^ The formation of this crystal habit can be attributed to the combination of solvent evaporation, the presence of ethanol in the reaction medium, and the interactions between organic ligands, which promote supramolecular organization around multiple simultaneous growth centers.

For the structural determination of [Zn­(phen)­(maleate)­(H_2_O)]·H_2_O, a suitable single crystal with average dimensions of 0.380 × 0.317 × 0.276 mm^3^ was selected and analyzed using single-crystal XRD. The crystallographic data revealed that the sample has the empirical formula C_16_H_14_N_2_O_6_Zn, a molecular weight of 395.66 g/mol, and a calculated density of 1.646 g/cm^3^. At room temperature (301 K), the coordination compound structure was found to belong to the triclinic system, with space group

$P\mathrm{1̅}\left(C_{i}^{1}\right)$ , and contains two formula [Zn­(phen)­(maleate)­(H_2_O)]·H_2_O per unit cell (*Z* = 2). The lattice parameters obtained were *a* = 8.6886(2) Å, *b* = 9.3334(3) Å, *c* = 10.5256(3) Å, α = 78.9980(10) °, β = 86.6300(10) °, γ = 72.3020(10) °, and *V* = 798.22(4) Å^3^. Additional structural data and refinement details are presented in Table S1.

Figure 2(**a**) displays the morphology of the crystal [Zn­(phen)­(maleate)­(H_2_O)]·H_2_O, modeled under ideal temperature and pressure conditions for the generation of the crystalline reticule. The crystal habit exhibits a polygonal nature, forming a spherical structure in which 14 distinct crystallographic planes were identified: (01̅1̅) , (1̅1̅1̅) , (1̅01̅) , (01̅0) , (001̅) , (1̅1̅0) , (1̅00) , (100), (101), (110), (001), (111), (010), and (011). However, the lack of precise control over the slow evaporation rate during the nucleation process did not favor the uniform development of all the terminal planes listed. Instead, preferential growth occurred along specific crystallographic directions, leading to a structure that resembles a spherulitic phase. This suggests that kinetic factors during solvent evaporation play a critical role in defining the final crystal morphology.^47^

**Figure 2:**
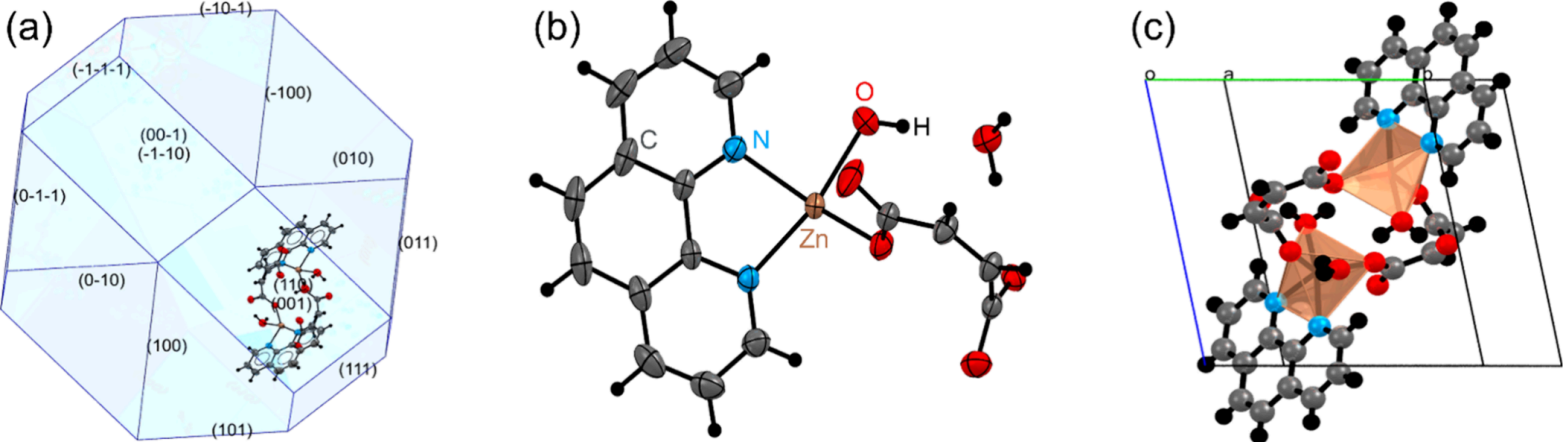
(**a**) Growth habit of [Zn­(phen)­(maleate)­(H_2_O)]·H_2_O crystal. (**b**) Asymmetric unit. (**c**) View of the coordination environment of the Zn­(II) center within the primitive unit cell, showing the four primary coordination bonds and the longer, intermolecular interaction that together form a distorted square pyramidal geometry in crystal lattice.

The molecular structure of [Zn­(phen)­(maleate)­(H_2_O)]·H_2_O is shown in Figure 2(**b**) using an ORTEP model with 50% thermal ellipsoids. The Zn^2+^ center appears to be tetracoordinated by three distinct ligands: one phen molecule, one maleate molecule, and one coordinated H_2_O molecule (equatorial positions), resulting in a slightly distorted square-planar geometry due to the distinct nature of the ligands. However, within the crystal lattice, this molecular unit does not exist in isolation. Supramolecular interactions lead to a fifth, longer coordination from an oxygen atom of a maleate ligand belonging to a neighboring molecule (Figure 2(**c**)). Consequently, the coordination sphere adopts a distorted square pyramidal geometry in the solid state, where the four closer atoms form the equatorial base and the longer intermolecular Zn···O contact occupies the axial position. This ‘4 + 1’ coordination is a key feature that links discrete molecules into a continuous supramolecular framework.

present the geometric parameters determined for the bond lengths and angles in the [Zn­(phen)­(maleate)­(H_2_O)]·H_2_O coordination compound. The bond distances around the Zn^2+^ center in unit cell confirm a distorted square pyramidal geometry, as evidenced by the variation in coordination bond lengths. The Zn–N bonds, involving the N atoms of the phen ligand, measure 2.1508(11) Å and 2.1187(10) Å, while the Zn–O bonds show greater variability: 2.0289(9) Å for the neighboring maleate ligand, 1.9769(9) Å for the directly coordinated maleate, and 2.1165(10) Å for the coordinated H_2_O molecule. These differences in bond lengths are attributed to the physicochemical properties of the coordinating ligands, such as molecular volume, polarity, and atomic composition, which collectively contribute to a steric hindrance around the metal center.^48,49^ This steric environment influences the spatial arrangement of the ligands and reinforces the observed distortion.

Additionally, it is important to highlight that the bond angle formed between the Zn center and the O and N atoms (originating from the neighboring maleate and the phen ligand) is 90.75(4)°, indicating a nearly orthogonal spatial arrangement. A similar value was observed for the angle formed between the metal center and the coordinated H_2_O and phen ligands, further supporting the distorted square pyramidal geometry of the coordination sphere. Such geometric features are consistent with the supramolecular organization observed in the crystal lattice and play a fundamental role in stabilizing the hydrogen-bonding lattice within the structure.

The supramolecular organization of [Zn­(phen)­(maleate)­(H_2_O)]·H_2_O is governed by an intricate lattice of secondary interactions, predominantly hydrogen bonding and π-π stacking (Figure S2). The coordinated H_2_O molecule serves as a hydrogen bond donor, forming strong O–H···O interactions with carboxylate oxygen atoms of neighboring maleate ligands [O–H···O: d­(O···O) = 2.6223(16) Å, O–H···O = 147.5°]. Similarly, the free H_2_O molecule bridges adjacent coordination units through bifurcated hydrogen bonds with both maleate and phen ligands [O–H···O: d­(O···O) = 2.8002(18) Å; O–H···O = 169.5°]. These contacts, detailed in Table S4, collectively form a 3D hydrogen-bonding lattice that extends throughout the crystal lattice, providing the primary cohesive force for structural stability.

Complementing the hydrogen-bonding lattice, π-π stacking interactions between parallel phen ligands further stabilize the crystal packing. Adjacent phen rings adopt a face-to-face arrangement with centroid-to-centroid distances of approximately 5.273 Å, consistent with effective aromatic stacking. These π-π interactions propagate along crystallographic structure, generating aromatic layers that alternate with the hydrogen-bonded aquo-maleate regions. The synergy between hydrogen bonding (providing directional stabilization) and π-π stacking (contributing to dispersive cohesion) defines the supramolecular architecture observed in this coordination compound.

The phase purity of the bulk synthesized [Zn­(phen)­(maleate)­(H_2_O)]·H_2_O was confirmed by PXRD analysis and by the Rietveld refinement method (Figure S3­(a)). The experimental diffraction pattern showed excellent agreement with the pattern simulated from single-crystal data (calculated), with all major peaks corresponding to the calculated reflections. The absence of extra peaks confirms the high phase purity, homogeneity of the synthesized compound and validates the reproducibility of the synthesis method. The refined lattice parameters were *a* = 8.700(9) Å, *b* = 9.348(3) Å, *c* = 10.539(4) Å, α = 78.98(5)°, β = 86.70(6)°, γ = 72.33(4)°, and V = 801.69(6) Å^3^. Furthermore, the quality indicators R_wp_ = 5.35%, R_p_ = 3.90%, and S = 1.16 demonstrate the consistency between the single-crystal and powder phases.

The thermal behavior of [Zn­(phen)­(maleate)­(H_2_O)]·H_2_O was investigated by DSC analysis. As shown in Figure S3­(**b**), no thermal events occur up to 318.6 K, indicating the compound stability within this temperature range. However, above this point a broad endothermic event is observed between 318.6 and 378.2 K, associated with the dehydration of H_2_O molecules. At higher temperatures, additional physicochemical events are detected, corresponding to the melting and decomposition processes of the sample.

### 3.2 Void Analysis

The characterization of voids within the primitive unit cell of [Zn­(phen)­(maleate)­(H_2_O)]·H_2_O reveals fundamental aspects of its supramolecular organization and potential solid-state reactivity. The projection of these voids along the crystallographic *a*-*c* axes (Figure 3) highlights a continuous and interconnected distribution of free space within the crystal lattice. Quantitative analysis indicates that the total void volume within the unit cell is 83.51 Å^3^, corresponding to 10.46% of the overall cell volume (798.22 Å^3^). This is a significant proportion, typical of coordination structures incorporating bulky organic ligands and free H_2_O molecules, which often generate channels or interstitial cavities.^50^ The total surface area of these voids, mapped via an electron density isosurface defined at 0.002 a.u., amounts to 268.52 Å^2^. This substantial surface area suggests that the voids are not merely isolated hollow vacancies, but form an accessible and potentially permeable lattice.

**Figure 3:**
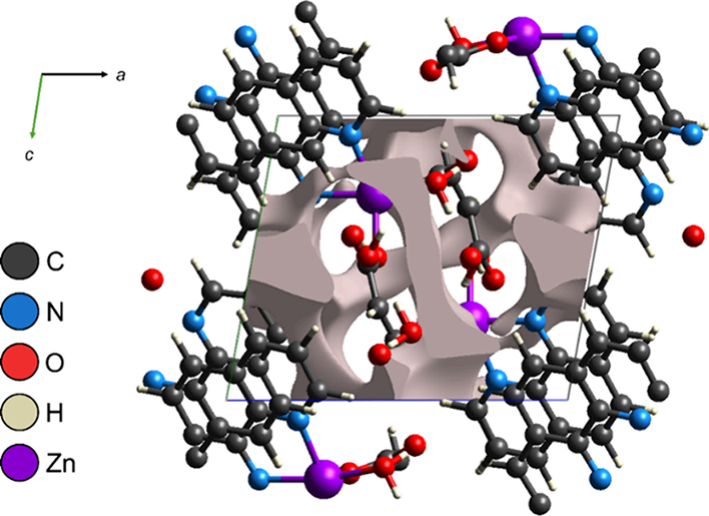
Projection of crystal voids viewed along the *a*-*c* crystallographic axes for the [Zn­(phen)­(maleate)­(H_2_O)]·H_2_O crystal.

The void shape indices, i.e., globularity of 0.344 and asphericity of 0.069, provide insights into the morphology of the isosurfaces.^51^ A globularity value near 1 (one) would indicate a perfectly spherical shape; however, the relatively low value of 0.344 confirms that the voids are highly nonspherical. The low asphericity (close to zero) supports this conclusion, indicating that the voids exhibit irregular, possibly channel-like geometries rather than isolated, rounded cavities. This morphology aligns with the formation of interconnected channels extending throughout the crystal, a common feature in supramolecular frameworks stabilized by hydrogen bonding and π-π stacking interactions.

The presence of this void lattice, occupying over 10% of the unit cell volume, has important implications for the properties of this coordination compound. Generally, structures with similar characteristics often exhibit inclusion capacity for small molecules, selective permeability **-** relevant for potential applications in separation or sensing, enhanced effective surface area **-** which may influence dissolution rates, surface reactivity, and even antibacterial activity by facilitating interactions with membranes or biological targets.^52^

The origin of these voids can be attributed to the molecular packing dictated by the bulky organic ligands (phen and maleate) and the presence of H_2_O molecules. The distorted geometry of the Zn^2+^ coordination sphere and the need to accommodate free H_2_O molecules within the lattice generate interstitial spaces. The intricate hydrogen-bonding lattice, detailed in **Section 3.1**, acts as a supramolecular “glue” that thermodynamically stabilizes this porous structure, ensuring its crystalline integrity. Overall, the void analysis confirms that [Zn­(phen)­(maleate)­(H_2_O)]·H_2_O is a crystalline material with significant porosity and channel-like void morphology. This structural feature may be crucial for modulating its physicochemical properties and biological activity.^53^

It is important to distinguish between the crystallographic voids characterized herein and the permanent porosity measured by gas adsorption techniques (e.g., BET analysis). Crystallographic voids represent interstitial spaces within the molecular crystal lattice, stabilized by supramolecular interactions and typically occupied by lattice solvent molecules. These voids are intrinsic to the packing arrangement but collapse upon desolvation, as removal of stabilizing solvent molecules disrupts the hydrogen-bonding lattice. In contrast, BET analysis is designed for materials with permanent, solvent-accessible porosity (e.g., metal–organic frameworks, zeolites), where structural channels persist after complete desolvation under vacuum.^54^ For molecular coordination compounds like [Zn­(phen)­(maleate)­(H_2_O)]·H_2_O, crystallographic void analysis using electron density isosurfaces is the standard and most appropriate method, providing molecular-level structural insights directly relevant to dissolution kinetics and bioavailability that are inaccessible through bulk gas adsorption measurements.^55^ The phase purity and crystallinity of the bulk material have been confirmed by PXRD with Rietveld refinement (Figure S3­(a)), validating that the single-crystal structure and its void distribution are representative of the entire sample.

### 3.3 Study of Noncovalent Bonds via Hirshfeld Surfaces

Hirshfeld surface analysis was employed to analyze the extensive lattice of noncovalent interactions that stabilize the supramolecular framework of the coordination compound [Zn­(phen)­(maleate)­(H_2_O)]·H_2_O. This theoretical tool provides a sophisticated, visual method for mapping the electron distribution associated with intermolecular interactions, moving beyond simple geometric analysis to offer a nuanced understanding of the cohesive forces present in a crystal, which are associated with the compound’s physicochemical behavior in biological environments.^56^ Figure 4 presents the Hirshfeld surfaces for the asymmetric unit, mapped according to key properties: *d*_norm_ in (**b**), *d*_i_ in (**c**), *d*_e_ in (**d**), shape index in (**e**), and curvedness in (**f**), all based on the asymmetric unit shown in (**a**).

**Figure 4:**
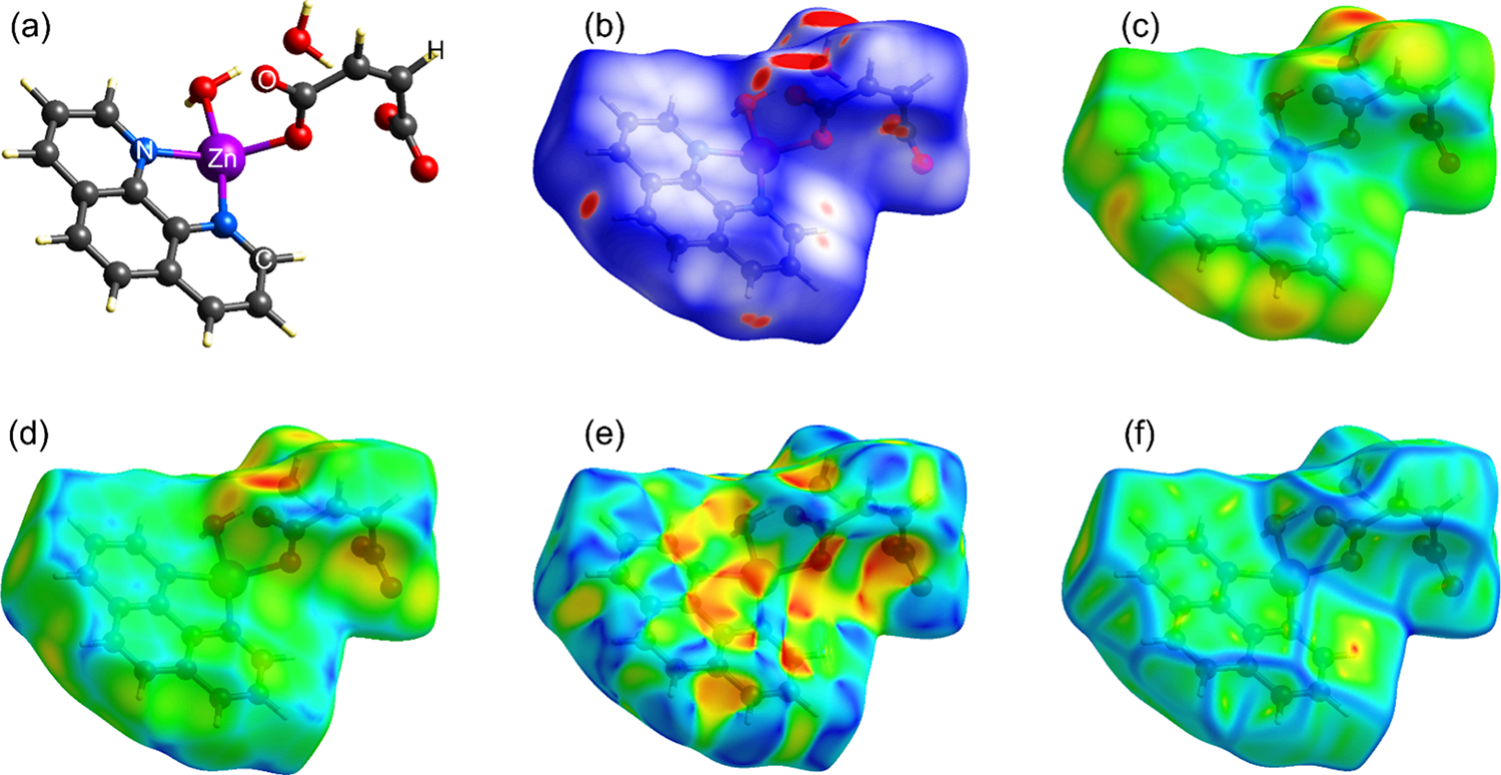
(**a**) Asymmetric unit of [Zn­(phen)­(maleate)­(H_2_O)]·H_2_O, and its Hirshfeld surfaces mapped according to (**b**) *d*_norm_, (**c**) *d*_i_, (**d**) *d*_e_, (**e**) shape index, and (**f**) curvedness.

The *d*_norm_ surface shown in Figure 4(**b**) serves as an immediate indicator of regions where intermolecular contacts are shorter than the sum of the *r*_vdW_. The prominent, intense red observed areas are related to the strongest hydrogen-bonding interactions. These are primarily located in the oxygen (O) atoms of both the coordinated H_2_O molecule (Zn–H_2_O) and the free H_2_O molecule, acting as prime acceptors, as well as in the O atoms of the carboxylate groups (COO^–^) of the maleate ligand, which function as both acceptors. The aromatic C–H portions of the phen ligand, engaging in weaker C–H···O interactions, are also evident. This continuous hydrogen-bonding lattice is a key determinant of crystal stability and its biological profile. For a potential drug candidate, such an extensive H-bonding lattice can moderate the release of the active species, potentially prevent accelerated release and contribute to a sustained antimicrobial effect.

The complementary *d*_i_ and *d*_e_ maps in Figure 4(**c**)-(**d**) allow for a more comprehensive analysis. The *d*_i_ map identifies the atoms within the molecule that are participating in close contacts, such as the H atoms of the H_2_O molecules, while the *d*_e_ map highlights the atoms from neighboring molecules that are penetrating the surface, such as the O atoms accepting these hydrogen bonds. The correlation between deep red-yellow regions on both maps confirms the directionality and strength of these O–H···O and O–H···N bonds, which form a continuous, 3D lattice throughout the framework. This extensive hydrogen-bonding scheme is the principal supramolecular “glue” responsible for the structural cohesion and stability inferred from crystallographic data. From a bioapplication standpoint, this high degree of hydration and H-bonding capacity suggests favorable interactions with aqueous biological fluids, potentially enhancing solubility and bioavailability, a crucial factor for antibacterial efficacy.

While hydrogen bonding provides the primary stabilization, the role of aromatic interactions is critical for the dense, layered packing observed. In this context, the shape index (Figure 4(**e**)) is exquisitely sensitive to π–π stacking.^57^ The presence of adjacent red triangles (concave regions) and blue triangles (convex regions) on the surfaces of the phen ligand is a fingerprint signature of face-to-face π–π stacking interactions. This alternating pattern suggests that the aromatic rings of adjacent molecules are intercalated, with the concave region of one ring accommodating the convex region of its neighbor. The curvedness map reinforces this finding (Figure 4(**f**)). Areas of low curvedness (flat, green regions) correspond to the planar phen rings engaged in stacking. The large, flat surfaces indicate areas of contact between aromatic systems, which contribute significantly to the overall lattice energy through dispersive forces. Notably, the planar, aromatic nature of the phen ligand, which facilitates this π-π stacking in the crystal, is the same structural feature that is known to promote intercalation into bacterial DNA (a proposed mechanism of action for this class of compounds).^4,5^ Thus, the crystal packing revealed by Hirshfeld surfaces directly mirrors the supramolecular recognition features that could be operative at the biological target site.

The full 2D fingerprint plot, shown in Figure S4, derived from the Hirshfeld surfaces, provides a quantitative breakdown of all interaction contributions. The analysis reveals that H···H contacts account for 33.2% of the total surface area, representing van der Waals interactions arising from the close packing of aliphatic and aromatic hydrogen atoms. These contacts fill interstitial voids and contribute significantly to the overall crystal density and structural compactness. The second most dominant interactions are H···O/O···H contacts (30.6%), confirming the fundamental role of hydrogen bonding in the supramolecular framework. This quantitative value, still demonstrates that approximately one-third of all intermolecular surface contacts involve H···O/O···H interactions, validating the presence of an extensive hydrogen-bonding lattice. The prevalence of these interactions is particularly relevant for biological applications, as they facilitate the compound’s solvation in aqueous media and may enhance its interaction with polar biological membranes and target sites.

H···C/C···H contacts constitute 18.3% of the surface interactions, representing a combination of weak C–H···π interactions and contacts at the edges of aromatic π-stacking regions. These interactions contribute to the stabilization of the phen ligand arrangement within the crystal lattice and may influence the ability to interact with aromatic residues in biological macromolecules, such as nucleic acids or aromatic amino acids in protein binding sites. The presence of C···C contacts (9.0%) provides direct evidence of π-π stacking interactions between adjacent phen ligands. This contribution underscores the importance of aromatic stacking forces in directing the supramolecular assembly and reinforces the structural preorganization of the phen ligand for potential DNA intercalation.

Additionally, Zn···O/O···Zn interactions account for 3.6% of the surface contacts, reflecting the coordination bonds and secondary coordination sphere interactions involving the Zn^2+^ center. These contacts are crucial for maintaining the integrity of the metal coordination geometry and may influence the Lewis acidity of the zinc center, which could be relevant for its reactivity with biological nucleophiles. H···N/N···H contacts (3.6%) indicate hydrogen-bonding interactions involving the nitrogen atoms of the phen ligand, likely through C–H···N weak hydrogen bonds. These interactions contribute to the overall lattice stability and may facilitate molecular recognition events with nitrogen-rich biological targets.

Minor contributions are observed from C···N/N···C (0.6%), O···N/N···O (0.5%), O···O (0.4%), O···C/C···O (0.1%), and Zn···H/H···Zn (0.1%) contacts. While individually small, these interactions collectively contribute to the fine-tuning of the crystal packing and may represent specific directional contacts that lock the structure into its observed conformation. The minimal Zn···H/H···Zn contacts suggest limited direct metal–hydrogen interactions, consistent with the predominantly ligand-centered coordination environment.

#### 3.3.1 Comparative Analysis with Structurally Related Coordination Compounds

To contextualize the significance of the intermolecular interaction profile observed in [Zn­(phen)­(maleate)­(H_2_O)]·H_2_O, a comparative analysis with structurally related zinc coordination compounds reported in the literature was performed (Table 1). The compounds selected for comparison include zinc complexes featuring N-donor ligands (phen or bipyridine analogues) and various coligands, representing the closest structural analogues with available quantitative Hirshfeld surface data.

**Table 1:** Comparative Hirshfeld Surface Interaction Percentages for [Zn­(phen)­(maleate)­(H_2_O)]·H_2_O and Structurally Related Zinc Coordination Compounds from the Literature

	Contact percentage [ % ]					
Compounds	H···H	H···O/O···H	H···C/C···H	C···C	Notable contacts	References
[Zn(phen)(maleate)(H_2_O)]·H_2_O	33.2	30.6	18.3	9.0	Zn···O/O···Zn – 3.6%	This work
[Zn(bipyridine)(xanthate)_2_]	36.3	14.4	15.1	2.9	S···H/H···S – 24.7%	^58^
[Zn(oxadiazole-thiolate)_2_]_n_	19.2	minor	19.5	minor	S···H/H···S – 19.0%	^59^
[Zn(sulfamethoxazole)_2_(H_2_O)_2_]	11.6	16.0	minor	minor	Cl···H/H···Cl – 43.4%	^60^

Comparative data reveal distinctive features of the [Zn­(phen)­(maleate)­(H_2_O)]·H_2_O supramolecular organization and highlight the influence of counterions and ligand architecture on intermolecular interaction profiles. The H···H contact percentage (33.2%) falls within the typical range for neutral coordination compounds,^49^ reflecting moderate van der Waals packing efficiency. Notably, this value is lower than the highly hydrophobic [Zn­(bipy)­(xanthate)_2_] complex (36.3%),^58^ where bulky xanthate ligands dominate the crystal packing, but significantly higher than both the polymeric [Zn­(oxadiazole-thiolate)_2_]_n_ (19.2%)^59^ and the ionic [Zn­(sulfamethoxazole)_2_(H_2_O)_2_] complex (11.6%).^60^ The low H···H contribution in the sulfamethoxazole complex is directly attributable to the dominant presence of chloride counterions, which reduce H···H contacts in favor of Cl···H/H···Cl interactions (43.4%). This comparison underscores neutral coordination compounds like the [Zn­(phen)­(maleate)­(H_2_O)]·H_2_O exhibit fundamentally different packing motifs compared to ionic species.

The most striking distinction of [Zn­(phen)­(maleate)­(H_2_O)]·H_2_O lies in its H···O/O···H contribution (30.6%), which represents the highest hydrogen-bonding capacity among all compared compounds. This value is nearly double that of the sulfamethoxazole complex (16.0%), more than twice that of the xanthate analogue (14.4%),^58^ and substantially exceeds typical values for mononuclear zinc complexes. The low H···O/O···H percentage in [Zn­(sulfamethoxazole)_2_(H_2_O)_2_] reflects the competitive influence of Cl^–^ anions,^60^ which preferentially form Cl···H/H···Cl bonds (43.4%) and disrupt the H_2_O-based hydrogen-bonding lattice. In contrast, the [Zn­(phen)­(maleate)­(H_2_O)]·H_2_O compound elevated percentage directly reflects the synergistic contribution of coordinated and lattice H_2_O molecules combined with the carboxylate functionalities of the maleate ligand, which collectively create a dense, 3D hydrogen-bonding lattice. From a biological perspective, this extensive hydration sphere is particularly relevant, as it suggests enhanced solvation capacity in aqueous biological fluids (a key factor for drug absorption and bioavailability). Compounds with high percentages of H···O/O···H typically exhibit favorable dissolution profiles and membrane permeability, supporting the compound’s potential for pharmaceutical applications.^61^ The stark contrast with the ionic [Zn­(sulfamethoxazole)_2_(H_2_O)_2_] complex further emphasizes that the neutral,^60^ hydrogen-bonded framework of [Zn­(phen)­(maleate)­(H_2_O)]·H_2_O may promote superior aqueous solubility without reliance on charge-assisted dissolution.

The C···C interaction percentage (9.0%) is by far the highest in this comparative series, directly attributable to the extended aromatic system of the phen ligand and its optimal spatial arrangement. Both the sulfamethoxazole complex (minor C···C) and the xanthate complex (2.9%) exhibit minimal aromatic stacking due to the absence or small size of aromatic systems and disruption by bulky substituents or counterions. This pronounced π-stacking is particularly significant for biological activity, as phen-based complexes with C···C contributions above 8% have demonstrated enhanced DNA intercalation capabilities.^62,63^

The H···C/C···H contact contribution (18.3%) confirms that weak C–H···π interactions play a dominant stabilizing role in phen-containing systems, exceeding the sulfamethoxazole complex (minor).^60^ This suggests enhanced hydrophobic interactions facilitating membrane partitioning, essential for intracellular antibacterial activity. Furthermore, the negligible H···C/C···H in the [Zn­(sulfamethoxazole)_2_(H_2_O)_2_] complex reflects its ionic character,^60^ where Cl···H/H···Cl interactions (43.4%) dominate the interaction landscape. A unique feature of [Zn­(phen)­(maleate)­(H_2_O)]·H_2_O is the presence of Zn···O/O···Zn contacts (3.6%), characteristic of secondary coordination sphere interactions. The maleate oxygen atoms engage in weak axial interactions with neighboring zinc centers, contributing to 3D supramolecular cohesion without forming coordination polymers, potentially modulating solid-state stability and dissolution kinetics.

Overall, the comparative Hirshfeld analysis establishes that [Zn­(phen)­(maleate)­(H_2_O)]·H_2_O combines the highest hydrogen-bonding capacity (H···O/O···H - 30.6%) with the strongest aromatic stacking (C···C - 9.0%) and balanced van der Waals cohesion (H···H - 33.2%). This multimodal interaction profile, fundamentally different from ionic complexes or polymeric systems, directly correlates with promising biological activity and favorable pharmacokinetics. The stark contrast with [Zn­(sulfamethoxazole)_2_(H_2_O)_2_],^60^ where chloride dominates (43.4%), highlights the strategic advantage of neutral, H_2_O-rich coordination compounds for antibacterial applications. These insights validate the [Zn­(phen)­(maleate)­(H_2_O)]·H_2_O potential and provide design principles for optimization, as enhancing hydrogen-bonding donors could improve aqueous solubility, while modifying the aromatic system could tune DNA-binding affinity and selectivity.

#### 3.3.2 Structure–Property Implications of the Supramolecular Organization

The quantitative Hirshfeld surface analysis provides crucial insights into how the supramolecular organization of [Zn­(phen)­(maleate)­(H_2_O)]·H_2_O influences its physicochemical properties. The unusually high percentage of H···O/O···H interactions (30.6%) compared to structurally related compounds has direct implications for several key properties.

The extensive hydrogen-bonding capacity, derived from both coordinated and lattice H_2_O molecules combined with carboxylate functionalities, creates a tuned hydration sphere that facilitates solvation in aqueous media. Materials with high percentages of H···O/O···H interactions typically exhibit enhanced dissolution rates due to favorable interactions with water molecules. This structural feature suggests that the compound should demonstrate good aqueous solubility, which is essential for pharmaceutical applications requiring absorption in biological fluids.

Furthermore, the dominant contribution of hydrogen bonding provides additional cohesive forces that stabilize the crystal lattice. This extensive noncovalent lattice, complemented by van der Waals interactions (H···H: 33.2%), ensures a mechanical stability and resistance to structural degradation. The combination of strong directional hydrogen bonds with dispersive forces creates a thermodynamically stable framework, as supported by the thermal analysis showing no decomposition events up to 318.6 K.

While extensive hydrogen bonding promotes aqueous solubility, it simultaneously presents challenges for membrane permeation. The hydrophilic character indicated by the high H···O/O···H percentage suggests that passive diffusion across lipophilic biological membranes may be limited. However, the significant H···C/C···H contacts (18.3%) reflect the presence of hydrophobic regions that partially balance the overall polarity, creating an amphiphilic molecular surface. This balanced character is a hallmark of drug-like molecules capable of navigating both aqueous biological fluids and membrane environments.

The pronounced C···C interaction percentage (9.0%), the highest among compared coordination compounds, reflects the optimal spatial arrangement of the phen ligand for π-π stacking. This structural feature indicates that the aromatic system is well-organized and accessible, which is relevant for potential interactions with aromatic biomolecules such as nucleic acids or aromatic amino acid residues in protein binding sites. The planar, extended aromatic architecture of phen, evidenced by the shape index and curvedness analysis showing flat surfaces engaged in stacking, suggests preorganization for intercalation into planar molecular architectures.

The Zn···O/O···Zn interactions (3.6%) represent secondary coordination sphere contacts that stabilize the distorted square pyramidal geometry in the solid state. These relatively weak interactions suggest that upon dissolution, the coordination environment may exhibit some flexibility, potentially allowing for dynamic rearrangement when interacting with biological targets. This structural adaptability could facilitate induced-fit binding mechanisms with biomolecular receptors.

The interconnected channel-like cavities identified in **Section 3.2**, occupying 10.46% of the unit cell volume, combined with the extensive hydrogen-bonding lattice, have important implications for dissolution behavior. The voids represent regions of lower packing density that can facilitate solvent penetration into the crystal lattice. When combined with the high density of hydrogen-bonding sites, this microstructural feature suggests that the compound should exhibit favorable dissolution kinetics, with rapid transition from solid dosage form to dissolved species. The lattice H_2_O molecules occupying these voids serve dual roles. They stabilize the crystal structure through hydrogen bonding and act as leaving groups during dissolution, creating transient channels that accelerate solvent access to the crystal interior.

The comprehensive Hirshfeld surface analysis establishes that [Zn­(phen)­(maleate)­(H_2_O)]·H_2_O exhibits a multimodal interaction profile combining exceptional hydrogen-bonding capacity (30.6%), pronounced aromatic stacking (9.0%), and balanced amphiphilic character (H···C/C···H: 18.3%). These structural features collectively predict favorable aqueous solubility, controlled dissolution kinetics, solid-state stability, and potential for molecular recognition events involving both polar and aromatic biomolecular targets. The subsequent sections will explore how these structure-based predictions correlate with experimental-theoretical properties and biological activity.

### 3.4 Geometric, Thermodynamic, and Electronic Properties via Periodic-DFT Calculations

DFT-periodic calculations were performed to gain deeper insights into the geometric, thermodynamic, and electronic properties of the coordination compound [Zn­(phen)­(maleate)­(H_2_O)]·H_2_O. The relaxed primitive unit cell, optimized using the GGA-PBE functional, is depicted in Figure 5(**a**). The computed lattice parameters show good agreement with the experimental single-crystal XRD data (Table S5), confirming the reliability of the theoretical model. The slight deviations observed (within 3.2%) can be attributed to the neglect of van der Waals corrections in the standard GGA functional, which slightly underestimates dispersive interactions critical for supramolecular packing.^40^ The optimized structure maintains the triclinic symmetry (space group $P\mathrm{1̅}\left(C_{i}^{1}\right)$ ) and accurately reproduces the distorted square pyramidal coordination geometry around the Zn^2+^ center, as well as the extensive hydrogen-bonding lattice involving H_2_O molecules and COO^–^ groups.

**Figure 5:**
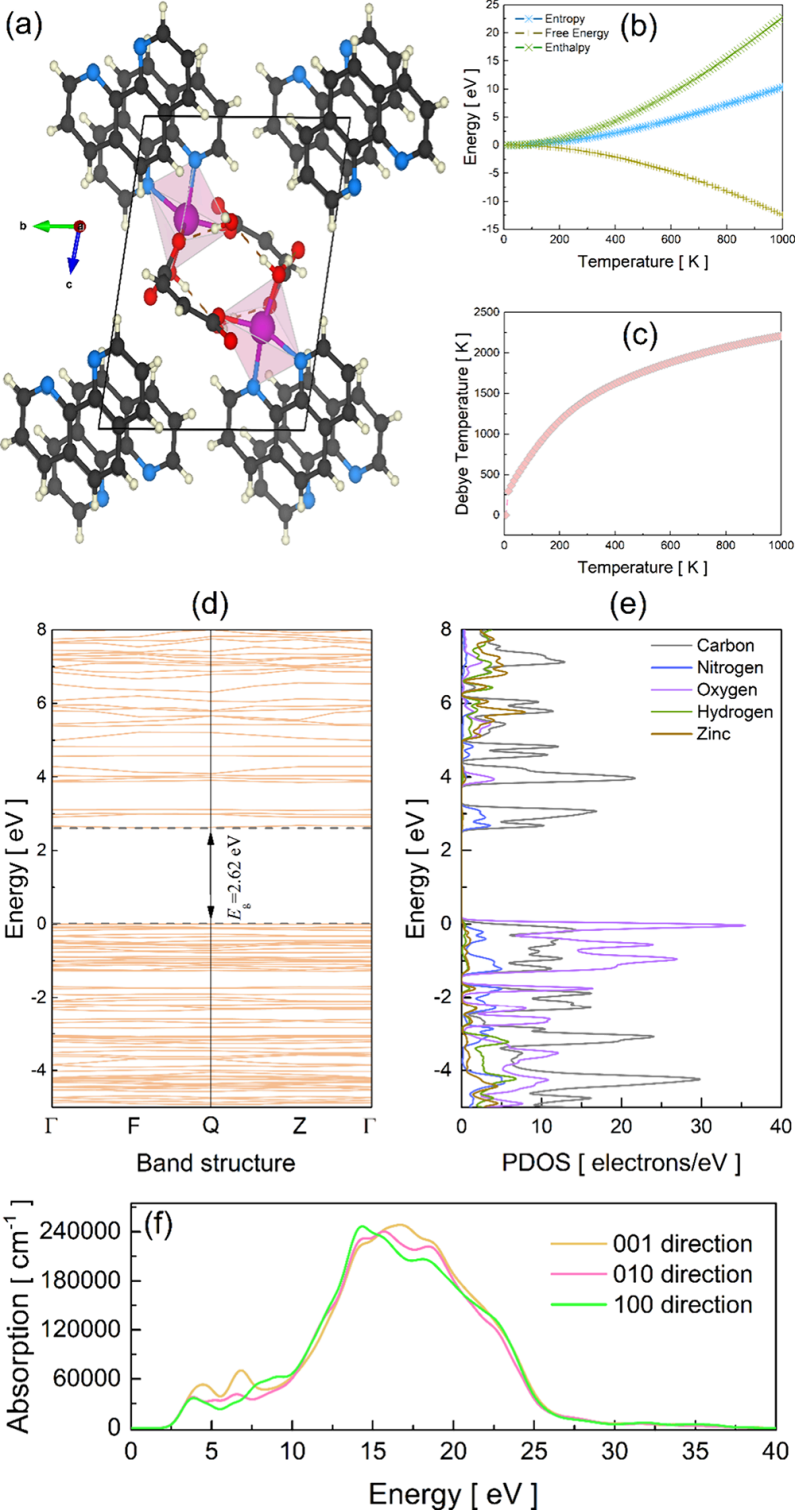
(**a**) Relaxed primitive unit cell of [Zn­(phen)­(maleate)­(H_2_O)]·H_2_O from the GGA-PBE method. (**b**) Thermodynamic variables. (**c**) Debye temperature estimated using CASTEP. (**d**) Band structure. (**e**) Contribution of elements around the gap electronic region from the PDOS. (**f**) Optical absorption.

The thermodynamic properties of the coordination compound [Zn­(phen)­(maleate)­(H_2_O)]·H_2_O, including enthalpy (*H*), entropy (*S*), and Gibbs free energy (*G*), were systematically evaluated as functions of temperature (0–1000 K), expressed in electron volts (eV), and are shown in Figure 5(**b**). The *H* variable increases gradually with temperature, reflecting the growing vibrational energy stored in the lattice. This behavior is attributed to the activation of low-frequency modes associated with the flexible organic ligands and H_2_O molecules. The *S* variable exhibits a less pronounced increase, indicating significant configurational and vibrational disorder at room temperature (≈1.2 eV). The *G* variable, calculated as *G* = *H* - T*S*, decreases steadily with increasing temperature, confirming the enhanced thermodynamic stability of [Zn­(phen)­(maleate)­(H_2_O)]·H_2_O under ambient conditions. This trend is consistent with the robust yet adaptable supramolecular framework, which efficiently dissipates thermal energy through its lattice of hydrogen bonds and π-stacking interactions.

The Debye temperature (Θ_*D*_), a fundamental parameter characterizing the vibrational properties and thermal conductivity of materials,^64^ was estimated from the computed phonon dispersion curves using CASTEP, based on Equation 2:

$$\Theta_{D}=\frac{\hbar }{k_{B}}{\left(6\pi^{2}N_{A}\rho /M\right)}^{1/3}v_{m}$$ 2

where *v*_*m*_ is the average sound velocity, ρ the density, *M* the molar mass, and *N*_*A*_ Avogadro’s number. In practice, the quasi-harmonic approximation approach uses phonon frequencies to compute thermodynamic functions and extract an effective Debye temperature at each temperature point. Debye Temperature is illustrated in Figure 5(**c**). The asymptotic value is the characteristic Debye temperature for this material, Θ_*D*_≈ 2.29 × 10^3^ K. This value is considered high and suggests a moderately stiff lattice. Such data imply that phonon-mediated thermal transport is limited, which may influence the thermal stability and reactivity of the coordination compound.

The electronic band structure and projected density of states (PDOS) provide electronic insights into the conductive and optical properties of the compound. As shown in Figure 5(**d**), the compound exhibits a direct energy gap (*E*_g_) of approximately 3.45 eV at the Q-point, characteristic of an effective electronic gap. The valence band maximum (*E*_g_ < 0) and conduction band (*E*_g_ > 0) minimum are both dominated by contributions from the phen and maleate ligands, with minimal involvement of Zn states near the Fermi level. This suggests that the electronic properties are primarily ligand-centered, which is consistent with the closed-shell *d* ^10^ configuration of Zn^2+^.

The PDOS graph in Figure 5(**e**) further elucidates the elemental contributions near the band gap. Zinc (Zn) atoms exhibit pronounced contributions in the deeper valence region, likely associated with *d*-orbital states. Oxygen (O) displays intense peaks throughout the valence band, consistent with its high electronegativity and strong bonding character. Nitrogen (N) shows a similar distribution to O atoms near the top of the valence band, reflecting its comparable electronegativity and role in bonding. In contrast, carbon (C) contributes more modestly in this region, while hydrogen (H) exhibits negligible participation, as expected due to its limited electronic states.^62^ A detailed orbital-resolved analysis is presented in Figure S5.

The orbital-resolved PDOS analysis (Figure S5) provides deeper insights into the electronic structure of the compound. For C atoms, the electronic states are predominantly derived from *p* orbitals, which dominate both the valence and conduction bands, while *s* orbitals contribute mainly at lower energies. O and N atoms exhibit a similar trend, with strong *p*-orbital contributions in the valence band, confirming their role in covalent bonding, whereas their *s* orbitals are localized at deeper energy levels. H atoms shows a negligible contribution. In contrast, Zn displays a significant presence of *d* orbitals in the deep valence region, characteristic of transition metals, along with minor contributions from *s* and *p* orbitals near the conduction band. These findings indicate that the valence band is primarily governed by *p* orbitals of O and N, while the conduction band involves *p* orbitals of C and *s*/*p* orbitals of Zn, with Zn *d* states playing an indirect role in the overall electronic structure. This orbital distribution is crucial for understanding the bonding nature and potential optical transitions in the material.

The optical absorption spectrum, calculated within the independent particle approximation, is presented in Figure 5(**f**). Optical absorption refers to the process by which a material absorbs photons from incident light, converting their energy into other forms, such as heat or electronic excitation. This phenomenon occurs when the energy of the incoming photons matches the energy difference between two electronic states in the material, typically between the valence and conduction bands. Furthermore, the absorption coefficient indicates the fraction of energy lost by the wave when it passes through the material, so that the intensity at a distance *x* from the surface is assumed by

$$I\left(x\right)=I\left(0\right)e^{-\eta x}$$ 3

where η represents the absorption coefficient, given by

$$\eta =\frac{2k\omega }{c}$$ 4

where *k* is the imaginary part of the complex refractive index. The absorption calculations were performed in a polarized configuration along the [100], [010], and [001] crystallographic directions.

The calculated absorption spectra (Figure 5(**f**)) exhibit a clear anisotropic response along [100], [010], and [001] directions. The optical anisotropy observed in the calculated spectra can be directly correlated with the structural features of the coordination compound [Zn­(phen)­(maleate)­(H_2_O)]·H_2_O. The nearly linear N1–Zn–O5 axis, which forms the most extended coordination direction, is oriented close to the crystallographic *c* axis. This alignment explains the enhanced absorption intensity for [001] polarization in the low-energy region (≤6 eV). Transitions in this range are likely dominated by metal-to-ligand charge transfer (MLCT) or ligand-to-metal charge transfer (LMCT) processes, which involve orbitals localized along the Zn coordination axis. The strong directional character of these orbitals results in a preferential interaction with the electric field component parallel to *c*.

The aromatic rings, which constitute the conjugated π-system of the ligand, exhibit a distinct orientation relative to the crystallographic axes. The normal to the phenyl ring plane is closer to the *a* axis, while the plane itself lies approximately parallel to the (*b*,*c*) plane. This geometry influences the polarization dependence of π–π transitions, which become more significant at higher energies. Consequently, in the vacuum ultraviolet (VUV) region (≥12 eV), the absorption maxima shift progressively from [100] to [010] and finally to [001], reflecting the combined effects of molecular orientation and the anisotropic distribution of transition dipole moments. Moreover, hydrogen bonding plays an additional role in modulating the optical response. The presence of strong O–H···O interactions creates supramolecular chains that propagate through the crystal lattice, introducing secondary anisotropy in the electronic structure.

The theoretical insights obtained from periodic-DFT calculations provide crucial understanding of the [Zn­(phen)­(maleate)­(H_2_O)]·H_2_O potential applications. The ligand-centered band gap of 3.45 eV and the minimal involvement of Zn states near the Fermi level explain the compound stability and moderate reactivity, which are desirable characteristics for a pharmaceutical agent where excessive reactivity could lead to toxicity. The anisotropic optical absorption behavior, particularly the enhanced intensity along the [001] direction, suggests potential for orientation-dependent interactions with biological membranes or directional charge transfer processes that could be exploited in photodynamic therapy applications. Furthermore, the thermodynamic stability predicted by the decreasing Gibbs free energy with temperature correlates with the experimental observation of compound stability under physiological conditions. The high Debye temperature (Θ_*D*_≈ 2.29 × 10^3^ K) indicates a stiff lattice that resists thermal degradation, supporting the compound’s potential for pharmaceutical formulations and for storage at room temperature.

### 3.5 Vibrational Spectroscopy and DFT Mode Assignments

The vibrational properties of the coordination compound [Zn­(phen)­(maleate)­(H_2_O)]·H_2_O were investigated using both Raman and FT-IR spectroscopies, combined with periodic-DFT calculations. This relation between experimental and theoretical approaches provides a comprehensive understanding of the lattice dynamics, molecular vibrations, and the nature of chemical bonds within the crystal structure. The experimental FT-IR and Raman spectra, recorded at room temperature, and the calculated spectra are presented in Figure 6(**a**) and 6(**b**), respectively, showing good agreement across the entire spectral range. The high degree of correlation validates the accuracy of the DFT-optimized structural model and allows for a suitable assignment of all observed vibrational modes, as detailed in Table S6.

**Figure 6:**
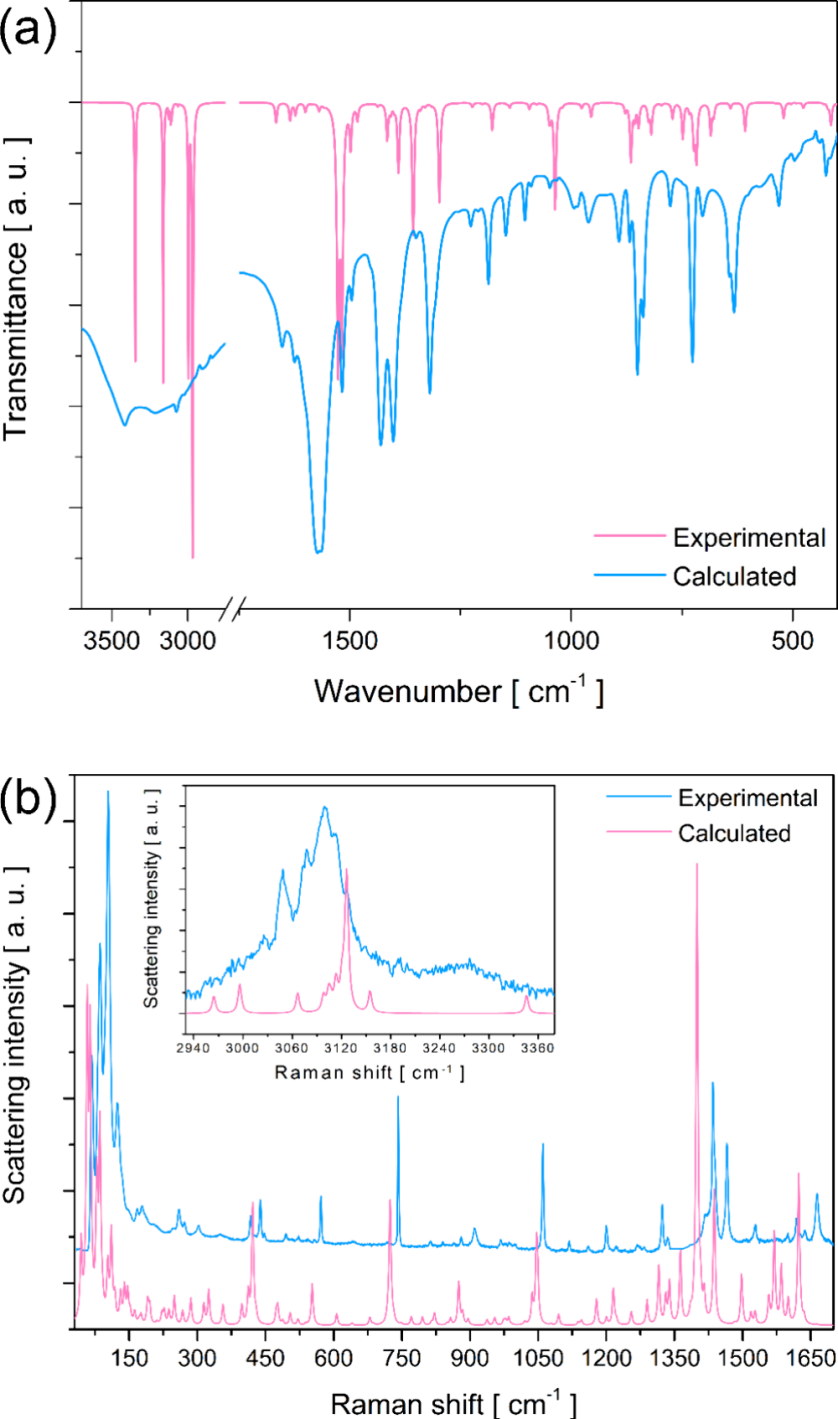
Experimental and calculated (**a**) FT-IR and (**b**) Raman spectra of the [Zn­(phen)­(maleate)­(H_2_O)]·H_2_O coordination compound.

The low-frequency region (below 200 cm^–1^) in Raman spectra is dominated by lattice modes, which are collective vibrations of the entire crystal lattice. The calculated modes in this region (64, 86, 103, 118, 147, 162, and 176 cm^–1^) are assigned to a complex mixture of translational motions of the free and coordinated water molecules (Tr­(H2O)), torsional modes involving the O–Zn–N coordination sphere (τ­(OZnN)), as well as the torsional (τ­(CCC)) modes of maleate and the ring deformation (δ­(ring)) modes of phen. The presence of these mixed modes underscores the strong coupling between the inorganic coordination sphere and the organic ligands, a direct consequence of the intricate hydrogen-bonding lattice that defines the supramolecular framework.

In the intermediate frequency range (200–1000 cm^–1^), the FT-IR and Raman spectra are characterized by a set of vibrations arising from internal deformations of the ligands and the metal coordination environment. Raman modes between 200–350 cm^–1^ (e.g., 210, 246, 267, 280, 314 cm^–1^) are primarily associated with torsional motions of the maleate anion (τ­(CCC)_maleate_) and deformations of the phen ring (δ­(ring)_phen_). The region from 350 to 640 cm^–1^ is heavily influenced by bending modes of the O–Zn–N coordination sphere (δ­(OZnN)), which are coupled with maleate torsions. The Raman bands at 476, 494, 510, 522, 543, 558, 571, and 643 cm^–1^, along with the FT-IR modes (412, 424, 438, 482, 496, 531, 540, 569, 600, and 642 cm^–1^) correspond closely to these calculated modes, confirming the specific vibrational signature of the distorted square pyramidal geometry around the Zn^2+^ center. Similarly, the low-wavenumber vibrational modes involving ligand–metal coordenation are characteristic of ternary phen-containing compounds, as also observed for the complexes [Nd­(phen)_2_(NO_3_)_3_],^49^ [Cu­(glycine)­(phen)­Cl]·3H_2_O,^65^ and [Ni­(phen)­(iso-leucine)_2_].^53^

The spectral window from 700 to 900 cm^–1^ is indicative of out-of-plane and bending vibrations of the C–H bonds (Φ­(CH)­maleate and δ­(ring)_phen_). The cluster of experimental Raman bands at 718, 797, 801, 813, 822, 840, 864, 880, and 910 cm^–1^, along with FT-IR bands at 703, 725, 775, 838, 851, 868, and 891 cm^–1^, aligns perfectly with the calculated values, demonstrating the sensitivity of Raman and FT-IR spectroscopies to the conformational details of the organic ligands.

The high-frequency region (950–1700 cm^–1^) contains the most intense bands, which are key fingerprints of the molecular structure. This region is dominated by stretching vibrations of the C–C and C–N bonds in the phen ring (ν­(CC)_phen_, ν­(CN)_phen_) and the carboxylate groups of the maleate ligand. The symmetric (ν_s_(COO)) and antisymmetric (ν_as_(COO)) stretching vibrations of the maleate anion are clearly identified in both Raman and FT-IR spectra. The ν_s_(COO) modes are observed in the range of 1200–1500 cm^–1^, while the characteristic ν_as_(COO) stretching appears as strong, well-defined bands between 1500 and 1670 cm^–1^. The positions and splitting of these carboxylate stretching are highly sensitive to the coordination mode (chelating vs bridging) and the strength of hydrogen bonding, providing further evidence for the ligand’s role in stabilizing the crystal structure.

In the high wavenumber region (2900–3200 cm^–1^), C–H stretching vibrations are observed as a series of closely spaced bands in both Raman (2961, 2994, 3048, 3078, 3099, 3112, 3126, 3149, 3191, and 3200 cm^–1^) and FT-IR (2836, 2901, 3068, 3223 cm^–1^) spectra, attributed to the aromatic bonds of the phen ligand (ν­(CH)_phen_). These vibrational modes have also been observed in other coordination compounds featuring the phen ligand, such as [Cu­(phen)­(methionine)­(H_2_O)]­Cl·1.5H_2_O^5^ and [Cu­(phen)­(tyrosine)­(H_2_O)].^66^ Additionally, a broad and intense band centered around 3222 cm^–1^ in Raman and 3409 cm^–1^ in FT-IR is assigned to the O–H stretching motions (ν­(OH)) of the coordinated H_2_O molecules. The breadth and lower wavenumber of these bands, compared to free H_2_O molecules, provide clear spectroscopic evidence of extensive hydrogen bonding involving these H_2_O molecules, as previously inferred from crystallographic and Hirshfeld surface analyses. The detailed mode assignments reveal a complex vibrational landscape where modes are highly coupled, reflecting the strong interplay between the metal center, coordinating and free ligands, and the pervasive supramolecular lattice of hydrogen bonds that governs the structural ordering.

The minor discrepancies observed between the experimental and theoretical Raman and FT-IR spectra can be primarily attributed to the fact that the periodic DFT calculations describe an ideal crystal at absolute zero (0 K), whereas the experimental measurements were performed at room temperature. Under these conditions, thermal expansion of the lattice and the population of higher vibrational energy levels lead to band broadening and slight shifts compared to the calculated harmonic frequencies.^67^ Despite these inherent effects of the theoretical model, the overall agreement between the experimental and calculated spectra is excellent, validating the optimized structural model and enabling reliable assignment of the vibrational modes.

### 3.6 Antibacterial Activity Studies

The biological activity evaluation of the novel coordination compound [Zn­(phen)­(maleate)­(H_2_O)]·H_2_O reveals a distinct and structure-dependent antibacterial activity profile. As presented in Table 2, the compound exhibits a MIC of 1000 μg/mL against the Gram-positive *Streptococcus mutans*. In contrast, no antibacterial activity was observed against the Gram-negative *Escherichia coli*. Representative images of the 96-well microdilution plates showing the colorimetric resazurin-based viability assessment are provided in Figure S6, and the corresponding dose–response curves demonstrating the concentration-dependent antibacterial effect are presented in Figure S7. The differential activity is a critical outcome, highlighting the compound’s selectivity and providing initial insights into its mechanism of action. Furthermore, the positive control antibiotic Gentamicin showed a much greater potency, with MIC values of 0.24 μg/mL and 1.95 μg/mL against *S. mutans* and *E. coli*, respectively.

**Table 2:** MIC Values of the [Zn­(phen)­(maleate)­(H_2_O)]·H_2_O Crystal, Precursor Compounds, and Gentamicin against the Bacteria *Streptococcus mutans* and *Escherichia coli*

Compound/Control	*Streptococcus mutans* [ μg/mL ]	*Escherichia coli* [ μg/mL ]
[Zn(phen)(maleate)(H_2_O)]·H_2_O	1000	No inhibition
Gentamicin (positive control)	0.24	1.95
ZnCl_2_	>1000 (inactive)	>1000 (inactive)
Phen	500	>1000 (inactive)
Maleate	>1000 (inactive)	>1000 (inactive)
DMSO (5% v/v)	No inhibition	No inhibition

To confirm that the observed activity originates from the intact coordination compound, control experiments were performed with individual components (Table 2). Free phen exhibited moderate activity against *S. mutans* (MIC = 500 μg/mL), consistent with its known antibacterial properties.^11,68^ However, neither ZnCl_2_ nor maleate showed activity at the tested concentrations (MIC > 1000 μg/mL), confirming that metal complexation modulates the biological response. The DMSO vehicle control (up to 5% v/v) showed no inhibitory effect.

The moderate antibacterial potency and selectivity for Gram-positive bacteria can be attributed to fundamental differences in bacterial cell envelope architecture. The thick peptidoglycan layer of Gram-positive bacteria is more permeable to the neutral coordination compound compared to the lipopolysaccharide-rich outer membrane of Gram-negative species.^4^ While these results indicate detectable antibacterial activity, the observed MIC values are substantially higher than those of conventional antibiotics. This preliminary screening serves primarily to establish proof-of-concept for biological activity and to identify structural features that could be optimized in future analogs. Significant enhancements in potency would be necessary through ligand modification, metal center variation, or nanoformulation strategies before this class of compounds could be considered for therapeutic development.

Overall, the observed antibacterial profile of [Zn­(phen)­(maleate)­(H_2_O)]·H_2_O can be directly correlated with its specific structural features. The distorted square-pyramidal geometry around the Zn^2+^ center, facilitated by the rigid chelating nature of the phen ligand, generates a molecular topology that influences membrane interactions. More importantly, the planar aromatic system of the phen ligand, evident from the extensive π–π stacking interactions observed in the crystal structure (**Section 3.3**), is structurally preorganized for potential DNA intercalation, providing a rational basis for the observed Gram-positive activity. At the same time, the hydrogen-bonding lattice involving both coordinated and free H_2_O molecules, composing 30.6% of the intermolecular contacts according to Hirshfeld surface analysis, regulates the compound’s hydration and dissolution behavior, directly impacting its bioavailability. The selective activity against Gram-positive *S. mutans* over Gram-negative *E. coli* thus reflects how these structural elements interact differently with the distinct frameworks of bacterial cell envelopes.

Although preliminary antimicrobial activity was detected for the [Zn­(phen)­(maleate)­(H_2_O)]·H_2_O, comprehensive cytotoxicity evaluation through MTT assays against normal mammalian cell lines (e.g., HEK-293, VERO) would be essential to establish the therapeutic index before considering this compound for pharmaceutical development. Such studies are crucial for validating the *in silico* toxicity predictions. The future direction lies in the rational design of drugs from this material by ligand modification to alter lipophilicity and by advanced formulation strategies to overcome the cellular permeability barriers and unlock its full potential as a broad-spectrum antimicrobial agent.

### 3.7 *In Silico* ADME and Druglikeness Parameters

As a preliminary assessment of the compound’s physicochemical profile and potential for structural optimization, *in silico* predictions were performed using the SwissADME platform (Table 3). It must be emphasized that these computational predictions serve solely as a rapid screening tool to identify structural features that could guide future analog design and do not substitute for experimental validation. Given the modest antimicrobial potency observed experimentally (MIC = 1000 μg/mL, **Section 3.6**), these results are interpreted as indicators of physicochemical properties rather than evidence of drug candidacy.

**Table 3:** ADME and Druglikeness Descriptors for the Coordination Compound [Zn­(phen)­(maleate)­(H_2_O)]·H_2_O

Physicochemical properties		Pharmacokinetics	
Molecular weight [g/mol]	395.66	CYP3A4 inhibitor	No
TPSA [Å^2^]	94.75	CYP2D6 inhibitor	No
Lipophilicity		Log *K*p^a^ [cm·s^–1^]	–7.49
Log *P*_o/w_ (SILICOS-IT)	–1.49	Druglikeness	
Hydrosolubility		Bioavailability score	0.56
Log *S* (SILICOS-IT)	2.74	Egan	Yes
Solubility [mg/mL]	7.25 × 10^–1^	Ghose	Yes
Class	Soluble	Lipinski	Yes
Pharmacokinetics		Veber	Yes
GI absorption	High	Muegge	Yes
P-gp substrate	Yes	Medicinal chemistry	
BBB permeant	No	PAINS	0 alert
CYP1A2 inhibitor	No	Lead-likeness	No; 1 violation: MW > 350
CYP2C9 inhibitor	No	Synthetic accessibility	3.48
CYP2C19 inhibitor	No	Brenk	3 alerts^b^

a Skin permeation.

b (*i*) Heavy metal, (*ii*) Michael acceptor 1, and (*iii*) polycyclic aromatic hydrocarbon 3.

The compound [Zn­(phen)­(maleate)­(H_2_O)]·H_2_O (molecular formula: C_16_H_14_N_2_O_6_Zn; MW = 395.66 g/mol) was predicted to exhibit high aqueous solubility (Log S = 2.74, classified as “soluble” with 0.725 mg/mL) and a hydrophilic character (Log P = −1.49). The topological polar surface area (TPSA = 94.75 Å) falls below the 140 Å threshold typically associated with favorable intestinal absorption.^69^ Computational analysis suggested high gastrointestinal (GI) absorption potential but predicted the compound as a P-glycoprotein (P-gp) substrate, which could reduce net bioavailability through efflux mechanisms. The model indicated no blood-brain barrier (BBB) penetration and no inhibition of cytochrome P450 isoforms (CYP1A2, 2C9, 2C19, 2D6, 3A4), the latter being favorable for minimizing drug–drug interaction risks.

Regarding compliance with established drug-likeness criteria, the compound satisfied all five major rules (Lipinski, Ghose, Veber, Egan, and Muegge), with a predicted bioavailability score of 0.56. However, it failed lead-likeness criteria due to molecular weight exceeding 350 g/mol. The synthetic accessibility score of 3.48 (scale: 1 = easy to 10 = difficult) indicates moderate synthetic complexity. Structural alerts were flagged by the Brenk filter for the presence of a heavy metal, a potential Michael acceptor, and a polycyclic aromatic hydrocarbon, though the absence of PAINS (Pan-Assay Interference Compounds) alerts is encouraging. It is important to note that zinc is an essential and biocompatible metal widely used in supplements and pharmaceuticals, mitigating concerns associated with the “heavy metal” alert.^7,70^

While these *in silico* predictions suggest that the physicochemical profile is within drug-like space and indicate potential for oral absorption, several critical limitations must be acknowledged. First, computational predictions have inherent uncertainties and cannot replace experimental pharmacokinetic studies. Second, the predicted properties reflect the compound in its current form, which exhibits only moderate antimicrobial activity. Third, the Brenk alerts, particularly regarding the Michael acceptor character, warrant experimental toxicity evaluation through MTT or similar cytotoxicity assays against mammalian cell lines to establish a therapeutic index. Fourth, the predicted P-gp substrate status suggests potential bioavailability challenges that would require formulation strategies or structural modifications to overcome.

The radar plot (Figure 7(**a**)) provides an intuitive visualization of the compound physicochemical properties. The red area in the center represents the optimal range for oral bioavailability. The plot shows that the compound fits perfectly within the boundaries for size, polarity, lipophilicity, solubility, and flexibility, confirming its promising potential drug-likeness character. The BOILED-Egg model (Figure 7(**b**)) graphically summarizes the absorption and BBB penetration predictions.^71^ The compound is located in the white region (yolk) of the plot, which accurately corresponds to the SwissADME predictions of high GI absorption and no BBB penetration. It is noteworthy that while the compound is not predicted to passively cross the BBB, its potential to reach the central nervous system could be facilitated through strategic formulation approaches, such as encapsulation in targeted nanocarriers, or via coadministration with known antibacterial agents that can enhance penetration for treating complex neurological infections.

**Figure 7:**
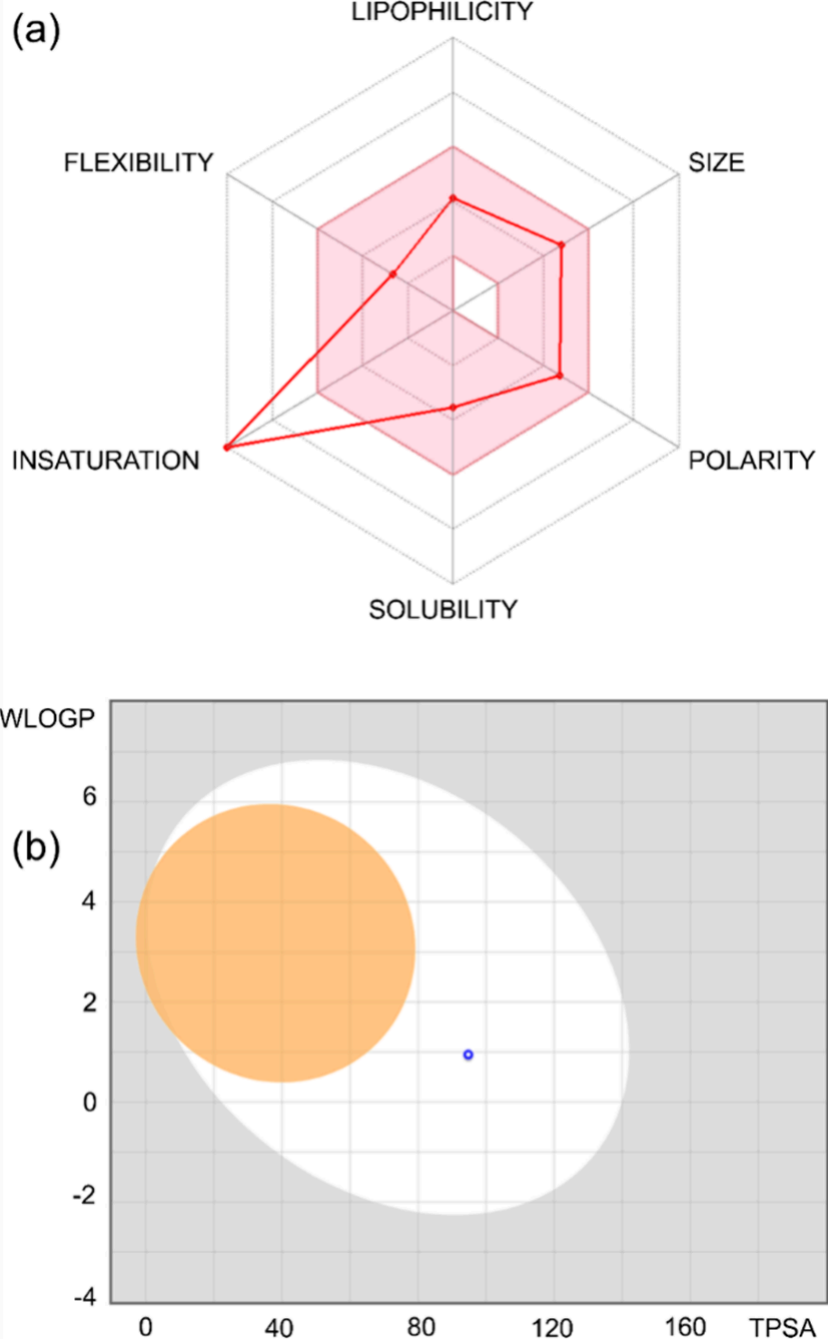
(**a**) Radar plot illustrating the physicochemical parameters calculated of [Zn­(phen)­(maleate)­(H_2_O)]·H_2_O, based on SwissADME. (**b**) BOILED-Egg graph for the coordination compound [Zn­(phen)­(maleate)­(H_2_O)]·H_2_O.

In summary, the *in silico* ADME analysis provides a preliminary physicochemical characterization that can inform future structural optimization efforts. The compliance with drug-likeness rules and predicted high GI absorption suggest that this framework could serve as a starting point for analog development, provided that substantial enhancements in antimicrobial potency are achieved through ligand modification, alternative metal centers, or advanced formulation approaches.

### 3.8 pH-Dependent Stability and Its Biological Implications

The stability profile of [Zn­(phen)­(maleate)­(H_2_O)]·H_2_O under different pH conditions provides crucial insights into its potential biomedical applications. As shown in Figure S8, the compound exhibited identical optical absorbance spectra at acidic (pH 3.7) and neutral (pH 7.0) conditions. The absorbance bands detected in the range of 300 to 360 nm correspond to internal electronic transitions of the phen ligand of the π–π* type.^5,62^ In contrast, at basic pH (10.5), complete precipitation occurred, preventing spectral measurements. It is also highlighted that no spectral variations were observed over a 24-h period, confirming the compound’ sustained stability in both acidic and neutral media.

The pH-dependent stability correlates well with the hydrogen-bonding lattice observed in the crystal structure (**Section 3.3**). The extensive hydration sphere and strong O–H···O interactions appear to induce structural stability in aqueous environments, particularly under acidic and neutral conditions. These findings have direct implications for the selection of the best administration route and formulation design, suggesting that, while intravenous or topical applications are promising, oral delivery would require additional stabilization strategies.

### 3.9 Computational Docking Studies

To gain preliminary insights into potential molecular targets, computational docking simulations were performed using AutoDock 4.2. The compound showed binding affinity to DNA (PDB ID: 1BNA, ΔG = −6.1 kJ/mol) and a *Streptococcus mutans* enzyme (PDB ID: 9CY9, ΔG = −7.4 kJ/mol), with the latter exhibiting stronger interaction through hydrogen bonds with GLN A:225 and SER A:70, and electrostatic interactions with ARG residues (detailed interaction maps in Supporting Information, Figure S9­(a,b). While these computational results suggest potential enzyme inhibition as a mechanism contributing to the observed antibacterial activity, they remain speculative given the moderate experimental potency (MIC = 1000 μg/mL). Experimental validation through enzymatic assays, DNA binding studies (e.g., UV–vis titration, circular dichroism), and cellular uptake experiments would be necessary to confirm these computational predictions. Direct visualization of bacteria-drug interactions through field-emission scanning electron microscopy would provide valuable mechanistic insights into cell membrane disruption and could confirm the mechanism proposed here based on the docking studies.

## 4 Conclusions

This study reports the design, synthesis, and comprehensive characterization of a novel Zn­(II) coordination compound, [Zn­(phen)­(maleate)­(H_2_O)]·H_2_O, integrating experimental and computational approaches to establish structure–property-activity relationships. Single-crystal XRD analysis revealed a distorted square pyramidal geometry forming a supramolecular framework (triclinic, P1̅ ) stabilized by hydrogen bonding (H···O/O···H: 30.6%, highest among structurally related Zn­(II) complexes) and π-π stacking interactions (C···C: 9.0%). Periodic-DFT calculations confirmed a direct energy gap of 3.45 eV and thermodynamic stability, while vibrational spectroscopy (FT-IR and Raman) combined with DFT provided comprehensive mode assignments.

The compound demonstrated selective antibacterial activity against Gram-positive *Streptococcus mutans* (MIC = 1000 μg/mL) with no activity against Gram-negative *Escherichia coli*. Control experiments confirmed activity originates from the intact coordination complex rather than individual components. Molecular docking revealed specific enzyme binding (ΔG = −7.4 kJ/mol). Preliminary *in silico* ADME screening indicated compliance with drug-likeness rules and predicted favorable gastrointestinal absorption, though experimental validation would be essential for any pharmaceutical consideration.

Despite these promising features, critical limitations must be acknowledged. The antibacterial potency is modest compared to gentamicin (MIC = 0.24 μg/mL), and the lack of Gram-negative activity significantly restricts the therapeutic spectrum. These limitations indicate that the compound, in its current form, is not immediately suitable for clinical development. However, this work represents a valuable proof-of-concept for rational Zn-coordination compounds design.

Several strategic modifications emerge as essential future directions. Ligand functionalization through introduction of lipophilic substituents on the phen backbone could enhance membrane permeability against Gram-negative bacteria. Advanced nanoformulation strategies, including encapsulation in lipid nanoparticles or polymeric micelles, may overcome outer membrane barriers and potentially reduce effective MIC values by one to 2 orders of magnitude. Exploration of isostructural Cu­(II) and Ag­(I) analogs could leverage the optimized supramolecular framework while enhancing intrinsic antimicrobial activity. Additionally, synergistic combination therapy with conventional antibiotics may reveal potentiation effects. Critically, comprehensive toxicological validation through *in vitro* cytotoxicity assays and *in vivo* studies remains an absolute prerequisite before any preclinical consideration.

The principal contribution of this work lies in establishing a systematic, multidisciplinary framework integrating experimental synthesis, advanced characterization, computational modeling, and controlled microbiological evaluation. The quantitative structure–activity relationships demonstrated here, particularly the correlation between supramolecular organization and selective biological activity, provide reproducible design principles for rational optimization of next-generation Zn-coordination compounds. While [Zn­(phen)­(maleate)­(H_2_O)]·H_2_O requires substantial optimization and rigorous toxicological validation before biomedical application, the systematic approach and mechanistic insights established herein contribute meaningfully to developing coordination chemistry-based strategies to combat bacterial resistance.

## Supplementary Material





## Notes

Image of a single crystal obtained by the slow solvent evaporation method; projection of a unit supercell of the coordination compound; PXRD pattern refined using the Rietveld method; 2D fingerprint plots (total and specific); PDOS contributions by atoms and orbitals; illustrative scheme of the antibacterial susceptibility assay against *Streptococcus mutans* and *Escherichia coli*; dose–response curves showing the effect of the complex on bacterial viability; UV–Vis absorbance spectra at different pH values; molecular docking poses; crystallographic parameters determined by single-crystal XRD; bond lengths and bond angles; hydrogen bonds; comparison between experimentally determined lattice parameters and those optimized via periodic-DFT calculations; analysis of experimental and calculated vibrational modes with their respective assignments (PDF)
XRD crystallographic information file obtained by single-crystal analysis (CCDC 2490001) (CIF)

## Notes

Deposition Number 2490001 contains the supplementary crystallographic data for this paper. These data can be obtained free of charge via the joint CCDC and Fachinformationszentrum Karlsruhe Access Structures service.
