# Supplementary material for: New Zinc(II) Coordination Compound with 1,10-Phenanthroline and Maleate: Comprehensive Structural Analysis, Periodic-DFT Calculations, and Evaluation of Biological Potential
Source: ACS Omega. 2026 Jan 22;11(4):5865–82. doi: 10.1021/acsomega.5c09973 (PMC12878713; doi:10.1021/acsomega.5c09973)
Supplement: Supplementary file 1 [file ao5c09973_si_001.pdf]

Supporting Information

A New Zinc(II) Coordination Compound with 1,10-Phenanthroline and Maleate: Comprehensive Structural Analysis, Periodic-DFT Calculations, and Evaluation of Biological Potential

João G. de Oliveira Neto[a,*], Jailton R. Viana[a], Anna R.P. Valerio[b], Otávio C. da Silva Neto[c], Luiz F. L. da Silva[d], Alejandro P. Ayala[e], Eliana B. Souto[f], Adenilson O. dos Santos[a], Rossano Lang[a,b,**]

[a] Center for Social Sciences, Health and Technology, Federal University of Maranhão (UFMA), 65900-410, Imperatriz, MA, Brazil

[b] Institute of Science and Technology, Federal University of São Paulo (UNIFESP), 12231-280, São José dos Campos, SP, Brazil

[c] Higher Education Institute of Southern Maranhão, Higher Education Unit of Southern Maranhão (UNISULMA), 65907-070, Imperatriz, MA, Brazil

[d] Criminalistics Institute, Scientific Police of Para, 68507-000, Marabá, PA, Brazil

[e] Department of Physics, Federal University of Ceara (UFC), 65455-900, Fortaleza, CE, Brazil

[f] UCD School of Chemical and Bioprocess Engineering, University College Dublin, Belfield, Dublin 4, D04 V1W8, Ireland

Corresponding authors: *joao.gon@ufma.br (J. G. de Oliveira Neto); **rossano.lang@unifesp.br (R. Lang).

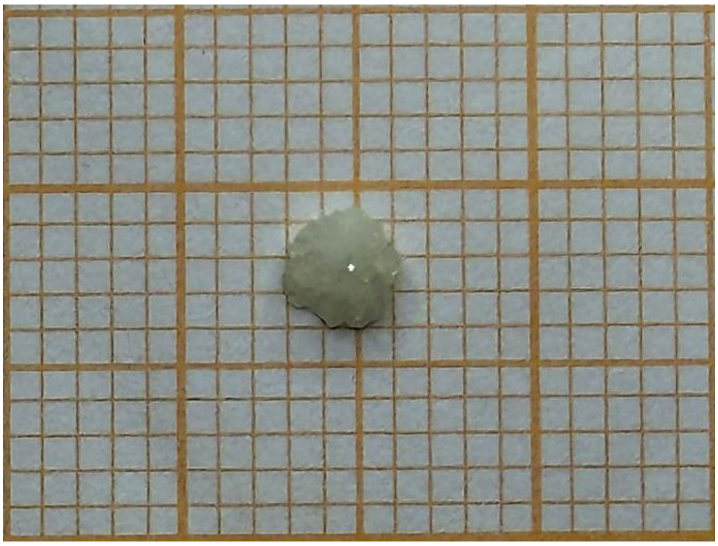

Figure S1. Photograph of a [Zn(phen)(maleate)(H_2O)]·H_2O crystal.

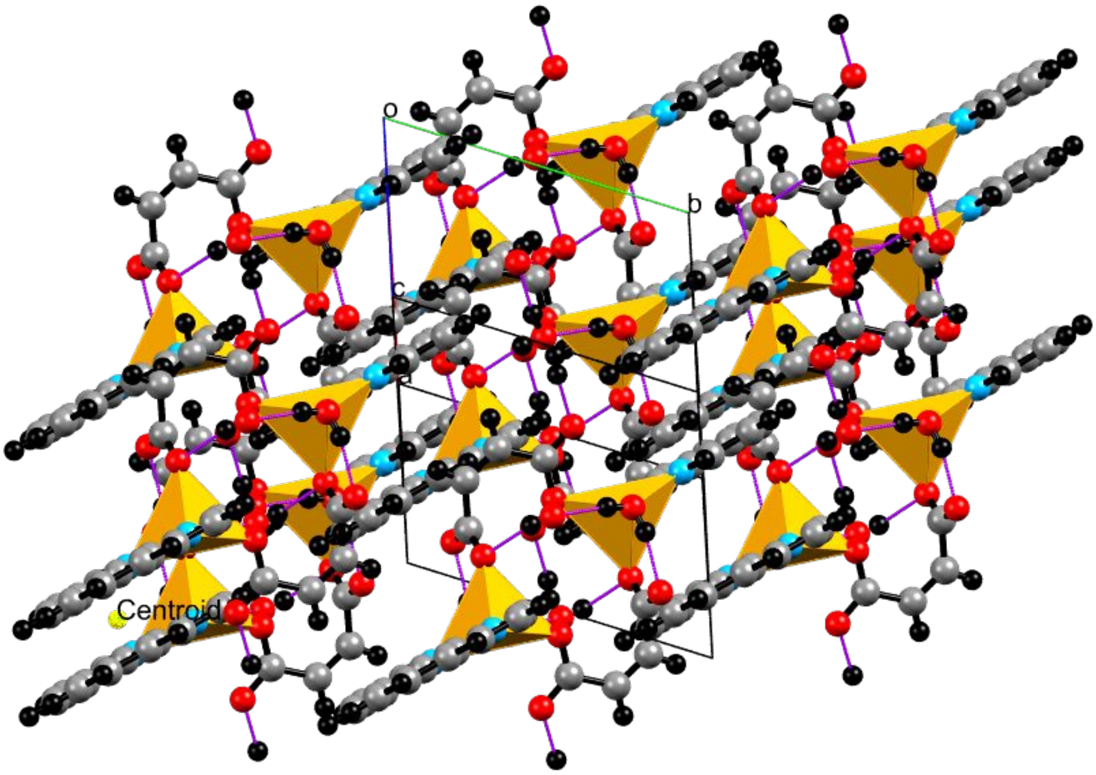

Figure S2. Projection of a unit supercell of the coordination compound [Zn(phen)(maleate)(H_2O)]·H_2O, illustrating the formation of the distorted pyramidal geometry (yellow polyhedra), the hydrogen bonds (purple dashed lines), and the π–π stacking interactions (centroid) between the monomers.

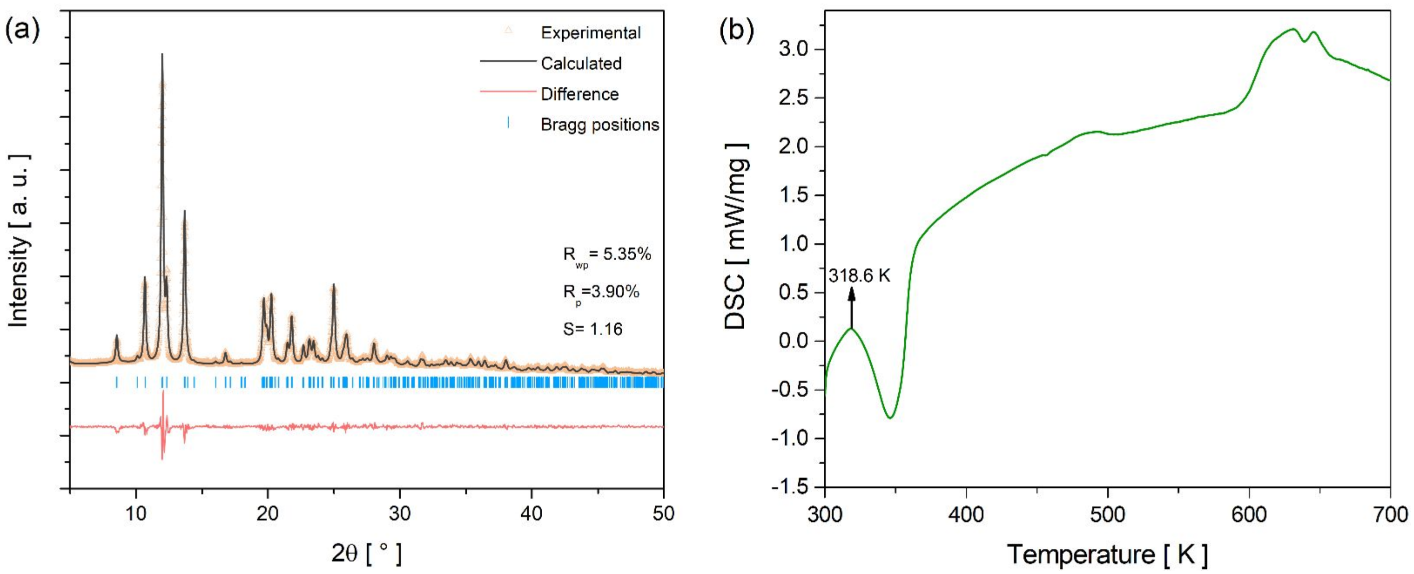

Figure S3. (**a**) Comparison of experimental PXRD pattern with simulated pattern from single-crystal data for [Zn(phen)(maleate)(H_2O)]·H_2O via Rietveld refinement method. (**b**) DSC curve.

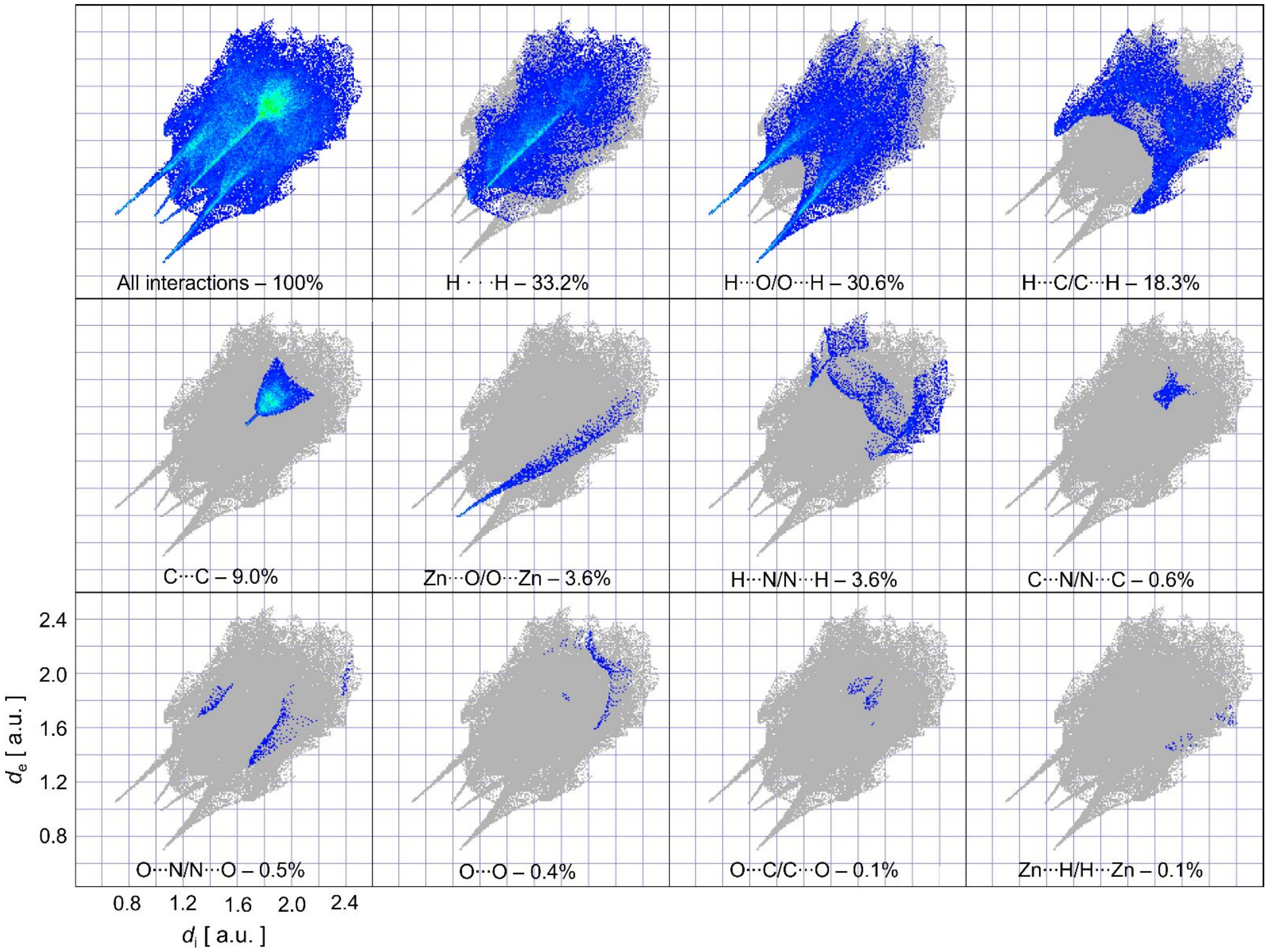

Figure S4. 2D fingerprint plots (total and specific) for [Zn(phen)(maleate)(H_2O)]·H_2O.

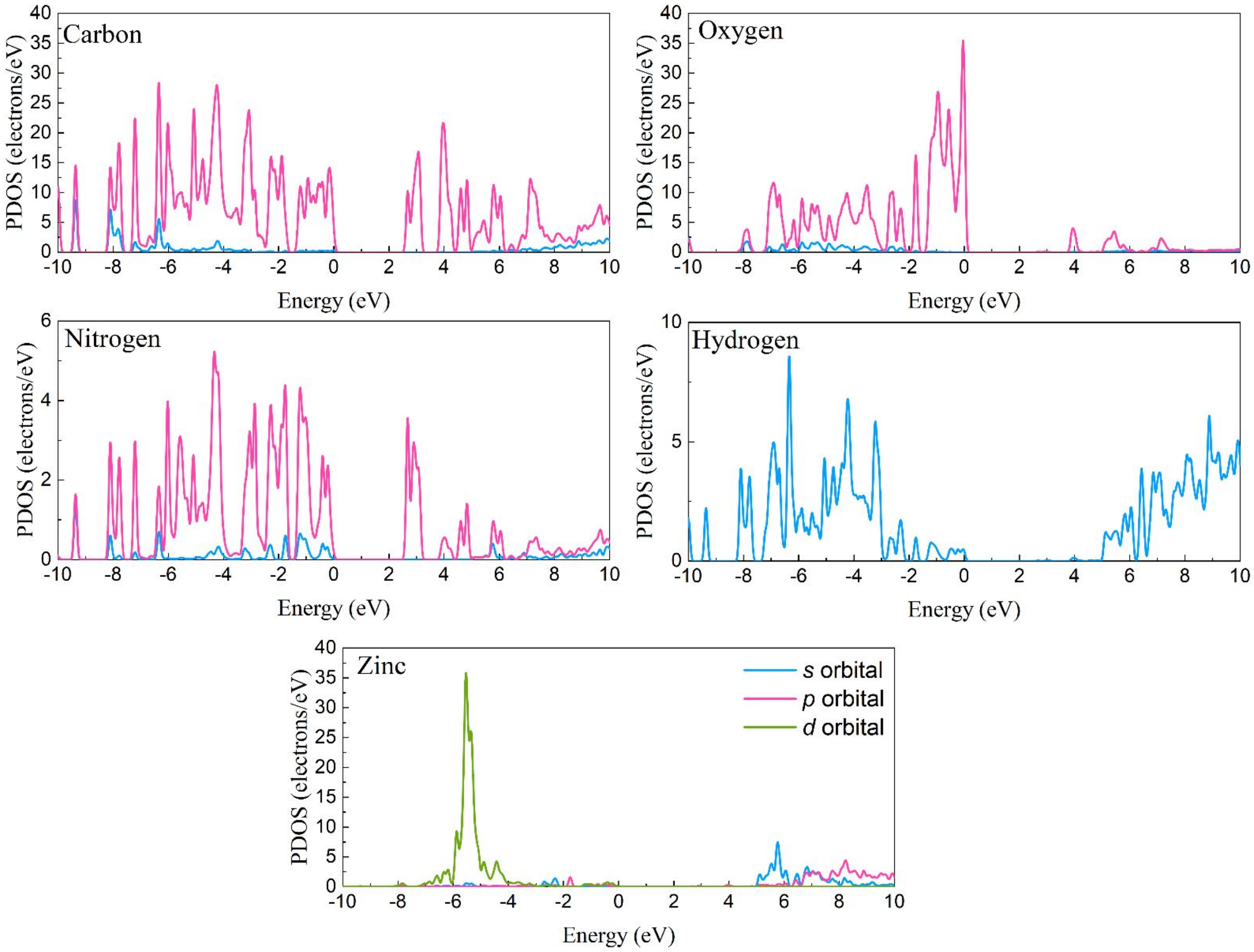

Figure S5. Orbital contributions around the electronic gap region through the PDOS.

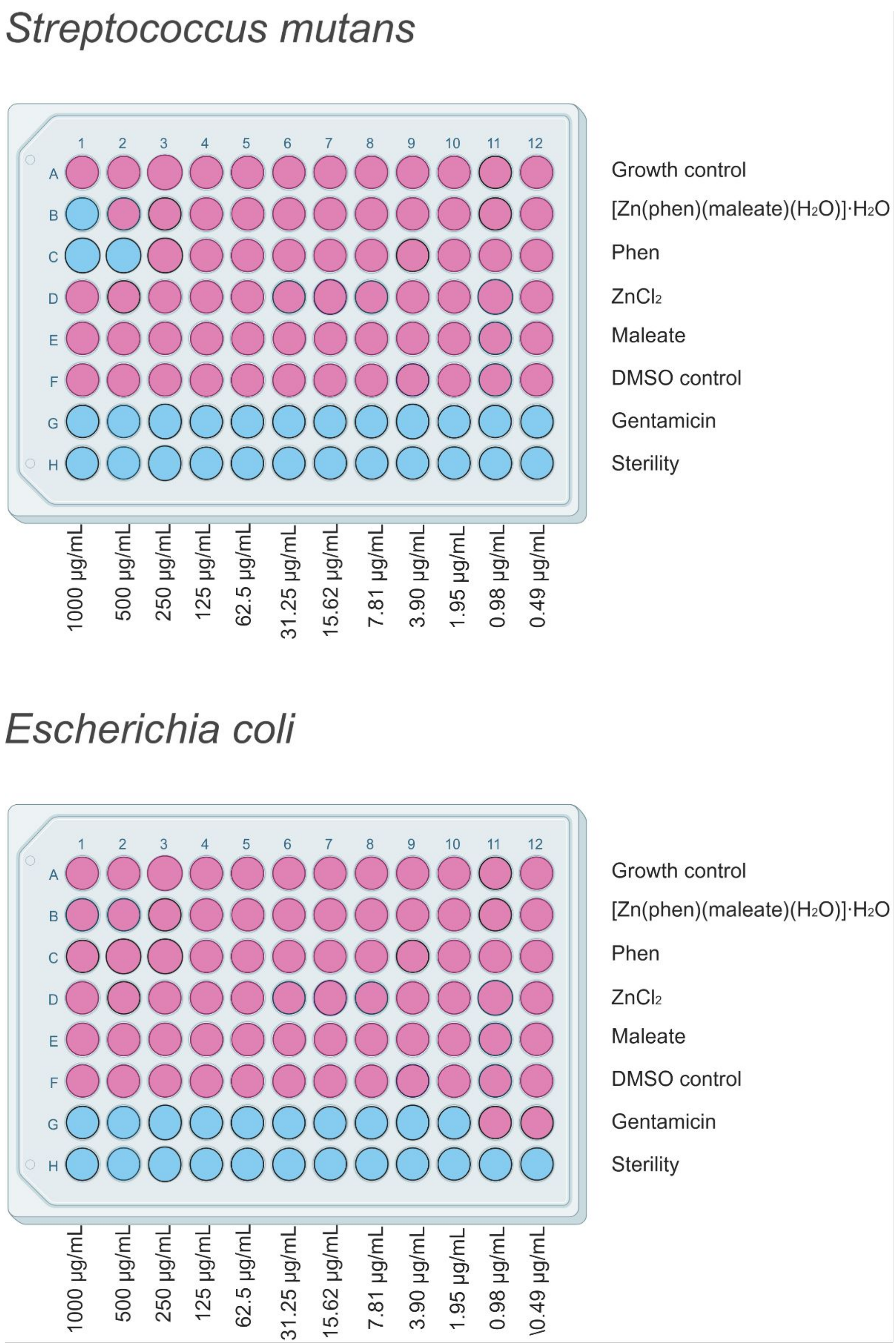

Figure S6. Representative antibacterial susceptibility assay (resazurin method) against *Streptococcus mutans* and *Escherichia coli*. Serial dilutions of the test compounds (1000–0.49 µg/mL) were arranged in columns 1-12. Blue wells indicate growth inhibition, while pink wells indicate bacterial growth. The compound [Zn(phen)(maleate)(H_2O)]·H_2O displayed a MIC of 1000 µg/mL against *S. mutans* only.

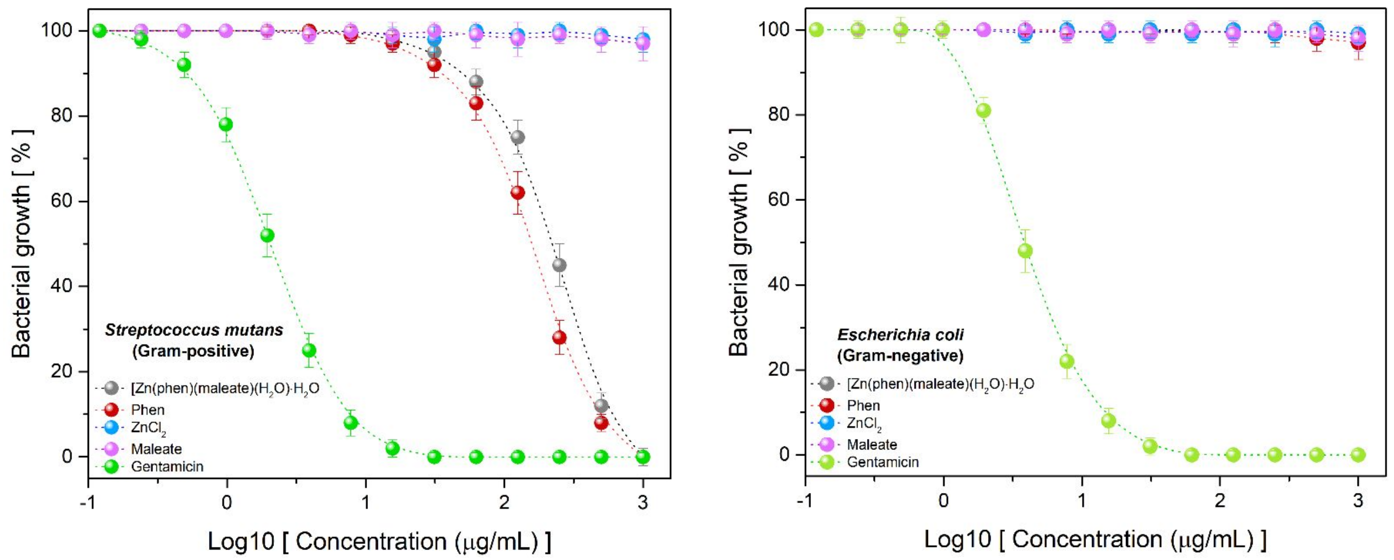

Figure S7. Dose–response curves illustrating the effect of [Zn(phen)(maleate)(H_2O)]·H_2O and its individual components on bacterial viability in *Streptococcus mutans* (Gram-positive) and *Escherichia coli* (Gram-negative).

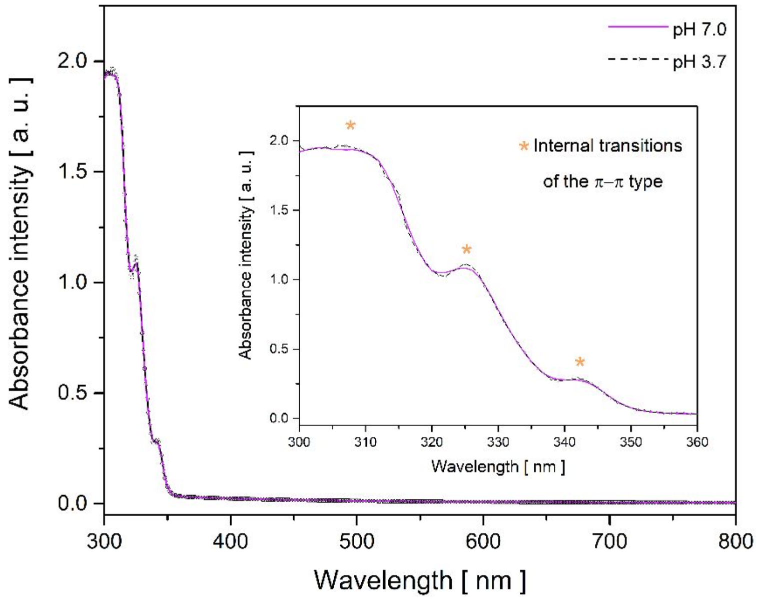

Figure S8. UV-Vis absorbance spectra of [Zn(phen)(maleate)(H_2O)]·H_2O at pH 3.7 and 7.0, recorded after 24 hours of dissolution.

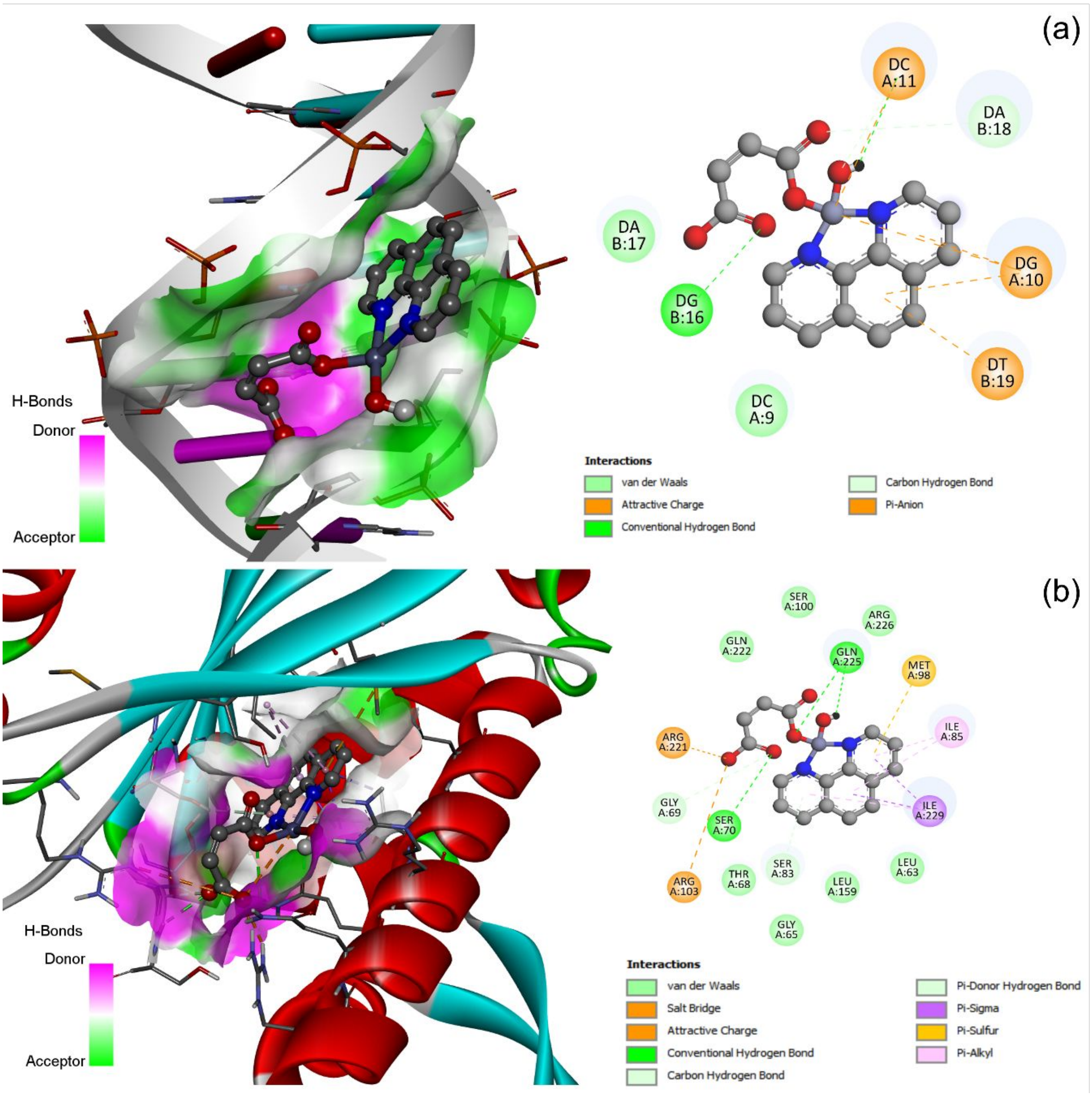

Figure S9. Molecular docking poses of [Zn(phen)(maleate)(H_2O)]·H_2O with (**a**) DNA (PDB ID: 1BNA) and (**b**) the *Streptococcus mutans* enzyme (PDB ID: 9CY9).

Table S1. Crystallographic and structure refinement data for the coordination compound [Zn(phen)(maleate)(H_2O)]·H_2O.

Chemical formula	[Zn(phen)(maleate)(H_2O)]·H_2O
Empirical formula	$C_{16}H_{14}N_2O_6Zn$
Formula weight [g/mol]	395.66
Temperature/K	301.00
Crystal system	triclinic
Space group	$P\bar{1}$
a [Å]	8.6886(2)
b [Å]	9.3334(3)
c [Å]	10.5256(3)
α [°]	78.9980(10)
β [°]	86.6300(10)
γ [°]	72.3020(10)
Volume [$Å^3$]	798.22(4)
Z	2
ρ_{calc} [g/cm^3]	1.646
M [mm^{-1}]	1.575
F(000)	404.0
Crystal size [mm^3]	0.380 × 0.317 × 0.276
Radiation	MoKα (λ = 0.71073)
2Θ range for data collection [°]	4.658 to 66.494
Index ranges	-13 ≤ h ≤ 13, -14 ≤ k ≤ 14, -16 ≤ l ≤ 16
Reflections collected	46759
Independent reflections	6135 [Rint = 0.0371, Rsigma = 0.0257]
Data/restraints/parameters	6135/0/230
Goodness-of-fit on F2	1.053
Final R indexes [I>=2σ (I)]	R_1 = 0.0270, wR_2 = 0.0659
Final R indexes [all data]	R_1 = 0.0364, wR_2 = 0.0725
Largest diff. [peak/hole / and $Å^{-3}$]	0.41/-0.50

CCDC 2490001 contains the supplementary crystallographic files for this work. These data can be obtained free of charge from CSD via https://bdec.dotlib.com.br/inicio_ asm/application/webcsd.

Table S2. Bond lengths for the crystal [Zn(phen)(maleate)(H_2O)]·H_2O determined via single-crystal XRD.

Atom	Atom	Length [Å]	Atom	Atom	Length [Å]
Zn1	O3[1]	2.0289(9)	C5	C4	1.4042(17)
Zn1	O1	1.9769(9)	C6	C7	1.4065(17)
Zn1	O5	2.1165(10)	C16	C15	1.4980(18)
Zn1	N2	2.1187(10)	C13	C14	1.4887(18)
Zn1	N1	2.1508(11)	C14	C15	1.3274(17)
O3	C16	1.2749(15)	C7	C8	1.435(2)
O1	C13	1.2664(17)	C7	C10	1.409(3)
O4	C16	1.2389(16)	C4	C9	1.430(2)
N2	C6	1.3561(17)	C4	C3	1.404(2)
N2	C12	1.3263(18)	C1	C2	1.400(2)
N1	C5	1.3549(15)	C12	C11	1.402(2)
N1	C1	1.3265(18)	C8	C9	1.344(3)
O2	C13	1.2404(16)	C2	C3	1.366(3)
C5	C6	1.4361(18)	C11	C10	1.360(3)

[1]1-*x*, 1-*y*, 1-*z*.

Table S3. Bond angles for the crystal [Zn(phen)(maleate)(H_2O)]·H_2O determined via single-crystal XRD.

**Atom**	**Atom**	**Atom**	**Angle [°]**	**Atom**	**Atom**	**Atom**	**Angle[°]**
O3[1]	Zn1	O5	93.38(4)	C7	C6	C5	119.10(13)
O3[1]	Zn1	N2	112.03(4)	O3	C16	C15	116.27(11)
O3[1]	Zn1	N1	90.75(4)	O4	C16	O3	126.25(12)
O1	Zn1	O3[1]	107.21(4)	O4	C16	C15	117.25(12)
O1	Zn1	O5	93.55(4)	O1	C13	C14	117.62(11)
O1	Zn1	N2	140.16(4)	O2	C13	O1	123.67(13)
O1	Zn1	N1	95.22(4)	O2	C13	C14	118.70(13)
O5	Zn1	N2	90.81(4)	C15	C14	C13	124.64(12)
O5	Zn1	N1	168.74(4)	C6	C7	C8	119.32(15)
N2	Zn1	N1	77.93(4)	C6	C7	C10	116.77(15)
C16	O3	Zn1[1]	127.38(8)	C10	C7	C8	123.92(14)
C13	O1	Zn1	112.11(8)	C14	C15	C16	126.61(11)
C6	N2	Zn1	113.89(8)	C5	C4	C9	119.31(15)
C12	N2	Zn1	127.87(10)	C3	C4	C5	117.31(13)
C12	N2	C6	118.23(12)	C3	C4	C9	123.38(14)
C5	N1	Zn1	113.21(8)	N1	C1	C2	122.50(14)
C1	N1	Zn1	128.35(9)	N2	C12	C11	122.45(16)
C1	N1	C5	118.38(12)	C9	C8	C7	121.37(14)
N1	C5	C6	117.10(11)	C3	C2	C1	119.44(15)
N1	C5	C4	122.82(13)	C10	C11	C12	119.57(16)
C4	C5	C6	120.08(12)	C11	C10	C7	119.85(14)
N2	C6	C5	117.76(10)	C8	C9	C4	120.82(15)
N2	C6	C7	123.14(13)	C2	C3	C4	119.55(14)

[1]1-*x*, 1-*y*, 1-*z*.

Table S4. Hydrogen bonds for the crystal [Zn(phen)(maleate)(H_2O)]·H_2O determined via single-crystal XRD.

D	H	A	d(D-H) [Å]	d(H-A) [Å]	d(D-A) [Å]	D-H-A[°]
O5	H5A	O4[1]	0.85	1.86	2.6223(16)	147.5
O5	H5B	O6	0.85	1.89	2.7314(16)	171.2
O6	H6A	O2[2]	0.85	1.96	2.8002(18)	169.5

[1]1-*x*, 1-*y*, 1-*z*; [2]-*x*, 1-*y*, 1-*z*.

Table S5. Optimized lattice parameters of the crystal [Zn(phen)(maleate)(H_2O)]·H_2O using the DFT-periodic calculations. The calculated data are compared to the experimental.

Lattice parameters	XRD	DFT	Difference	Difference in %
a [Å]	8.6886(2)	8.9629	0.2743	3.15
b [Å]	9.3334(3)	9.5882	0.2548	2.73
c [Å]	10.5256(3)	10.7889	0.2633	2.50
α [°]	78.9980(10)	78.5949	0.4031	0.51
β [°]	86.6300(10)	86.1917	0.4383	0.51
γ [°]	72.3020(10)	72.0618	0,2402	0.33

Table S6. Observed vibration mode analyses for the $[Zn(phen)(maleate)(H_2O)]\cdot H_2O$ crystal: calculated wavenumbers (ω_{cal}), experimental Raman modes (ω_R), experimental IR modes (ω_{IR}), irreducible representation (Irrep.), and their assignments.

ω_{cal} [cm^{-1}]	ω_R [cm^{-1}]	ω_{IR} [cm^{-1}]	Irrep.	Assignments†
64	68	-	Ag	$Tr(H_2O) + \tau(OZnN) + \tau(CCC)_{maleate} + \delta(ring)_{phen}$
86	86	-	Ag	$Tr(H_2O) + \tau(OZnN) + \tau(CCC)_{maleate} + \delta(ring)_{phen}$
103	104	-	Ag	$Tr(H_2O) + \tau(OZnN) + \tau(CCC)_{maleate} + \delta(ring)_{phen}$
118	124	-	Ag	$Tr(H_2O) + \tau(OZnN) + \tau(CCC)_{maleate} + \delta(ring)_{phen}$
147	146	-	Ag	$Tr(H_2O) + \tau(OZnN) + \tau(CCC)_{maleate} + \delta(ring)_{phen}$
162	167	-	Ag	$Tr(H_2O) + \tau(OZnN) + \tau(CCC)_{maleate} + \delta(ring)_{phen}$
176	178	-	Ag	$Tr(H_2O) + \tau(OZnN) + \tau(CCC)_{maleate} + \delta(ring)_{phen}$
210	206	-	Ag	$\tau(CCC)_{maleate} + \delta(ring)_{phen}$
246	244	-	Ag	$\tau(CCC)_{maleate} + \delta(ring)_{phen}$
267	259	-	Ag	$\tau(CCC)_{maleate} + \delta(ring)_{phen}$
280	271	-	Ag	$\tau(CCC)_{maleate} + \delta(ring)_{phen}$
314	303	-	Ag	$\tau(CCC)_{maleate} + \delta(ring)_{phen}$
356	349	-	Ag	$\delta(OZnN) + \tau(CCC)_{maleate} + \delta(ring)_{phen}$
413	-	412	Au	$\delta(OZnN) + \tau(CCC)_{maleate} + \delta(ring)_{phen}$
412	417	-	Ag	$\delta(OZnN) + \tau(CCC)_{maleate} + \delta(ring)_{phen}$
420	-	424	Au	$\delta(OZnN) + \tau(CCC)_{maleate} + \delta(ring)_{phen}$
421	437	-	Ag	$\delta(OZnN) + \tau(CCC)_{maleate} + \delta(ring)_{phen}$
430	-	438	Au	$\delta(OZnN) + \tau(CCC)_{maleate} + \delta(ring)_{phen}$
429	447	-	Ag	$\delta(OZnN) + \tau(CCC)_{maleate} + \delta(ring)_{phen}$
475	476	-	Ag	$\delta(OZnN) + \tau(CCC)_{maleate}$
476	-	482	Au	$\delta(OZnN) + \tau(CCC)_{maleate}$
488	494	-	Ag	$\delta(OZnN) + \tau(CCC)_{maleate}$
491	-	496	Au	$\delta(OZnN) + \tau(CCC)_{maleate}$
504	510	-	Ag	$\delta(OZnN) + \tau(CCC)_{maleate}$
521	522	-	Ag	$\delta(OZnN) + \tau(CCC)_{maleate}$
520	-	531	Au	$\delta(OZnN) + \tau(CCC)_{maleate}$
538	-	540	Au	$\delta(OZnN) + \tau(CCC)_{maleate}$
539	543	-	Ag	$\delta(OZnN) + \tau(CCC)_{maleate}$
552	558	-	Ag	$\delta(OZnN) + \tau(CCC)_{maleate}$
551	-	569	Au	$\delta(OZnN) + \tau(CCC)_{maleate}$
599	571	-	Ag	$\delta(OZnN) + \tau(CCC)_{maleate}$
599	-	600	Au	$\delta(OZnN) + \tau(CCC)_{maleate}$
641	-	642	Au	$\delta(OZnN) + \tau(CCC)_{maleate}$
640	643	-	Ag	$\delta(OZnN) + \tau(CCC)_{maleate}$
717	-	703	Au	$\delta(ring)_{phen} + \Phi(CH)_{maleate}$
717	718	-	Ag	$\delta(ring)_{phen} + \Phi(CH)_{maleate}$
724	-	725	Au	$\delta(ring)_{phen} + \Phi(CH)_{maleate}$
769	-	775	Au	$\delta(ring)_{phen} + \Phi(CH)_{maleate}$
771	797	-	Ag	$\delta(ring)_{phen} + \Phi(CH)_{maleate}$
795	801	-	Ag	$\delta(ring)_{phen} + \Phi(CH)_{maleate}$
815	813	-	Ag	$\delta(ring)_{phen} + \Phi(CH)_{maleate}$

822	822	-	Ag	$\delta(ring)_{phen} + \Phi(CH)_{maleate}$
826	-	838	Au	$\delta(ring)_{phen} + \Phi(CH)_{maleate}$
849	840	-	Ag	$\delta(ring)_{phen} + \Phi(CH)_{maleate}$
848	-	851	Au	$\delta(ring)_{phen} + \Phi(CH)_{maleate}$
857	864	-	Ag	$\delta(ring)_{phen} + \Phi(CH)_{maleate}$
865	-	868	Au	$\delta(ring)_{phen} + \Phi(CH)_{maleate}$
884	880	-	Ag	$\delta(ring)_{phen} + \Phi(CH)_{maleate}$
895	-	891	Au	$\delta(ring)_{phen} + \Phi(CH)_{maleate}$
896	910	-	Ag	$\delta(ring)_{phen} + \Phi(CH)_{maleate}$
956	-	960	Au	$\delta(ring)_{phen} + \nu(CO)_{maleate} + \nu(CC)_{maleate}$
954	966	-	Ag	$\delta(ring)_{phen} + \nu(CO)_{maleate} + \nu(CC)_{maleate}$
974	976	-	Ag	$\delta(ring)_{phen} + \nu(CO)_{maleate} + \nu(CC)_{maleate}$
978	980	-	Ag	$\delta(ring)_{phen} + \nu(CO)_{maleate} + \nu(CC)_{maleate}$
985	-	984	Au	$\delta(ring)_{phen} + \nu(CO)_{maleate} + \nu(CC)_{maleate}$
985	985	-	Ag	$\delta(ring)_{phen} + \nu(CO)_{maleate} + \nu(CC)_{maleate}$
992	-	993	Au	$\delta(ring)_{phen} + \nu(CO)_{maleate} + \nu(CC)_{maleate}$
992	995	-	Ag	$\delta(ring)_{phen} + \nu(CO)_{maleate} + \nu(CC)_{maleate}$
1020	1018	-	Ag	$\delta(ring)_{phen} + \nu(CO)_{maleate} + \nu(CC)_{maleate}$
1036	1025	-	Ag	$\delta(ring)_{phen} + \nu(CO)_{maleate} + \nu(CC)_{maleate}$
1046	1036	-	Ag	$\delta(ring)_{phen} + \nu(CO)_{maleate} + \nu(CC)_{maleate}$
1049	-	1048	Au	$\delta(ring)_{phen} + \nu(CO)_{maleate} + \nu(CC)_{maleate}$
1050	1060	-	Ag	$\delta(ring)_{phen} + \nu(CO)_{maleate} + \nu(CC)_{maleate}$
1095	1118	-	Ag	$\delta(ring)_{phen} + \nu(CO)_{maleate} + \nu(CC)_{maleate}$
1138	1159	-	Ag	$\delta(ring)_{phen} + \nu(CO)_{maleate} + \nu(CC)_{maleate}$
1094	-	1091	Au	$\delta(ring)_{phen} + \nu(CO)_{maleate} + \nu(CC)_{maleate}$
1138	-	1146	Au	$\delta(ring)_{phen} + \nu(CO)_{maleate} + \nu(CC)_{maleate}$
1178	-	1186	Au	$\delta(ring)_{phen} + \nu(CO)_{maleate} + \nu(CC)_{maleate}$
1178	1199	-	Ag	$\delta(ring)_{phen} + \nu(CO)_{maleate} + \nu(CC)_{maleate}$
1200	1210	-	Ag	$\nu(CC)_{phen} + \nu(CN)_{phen} + \nu_s(COO)_{maleate}$
1215	-	1211	Au	$\nu(CC)_{phen} + \nu(CN)_{phen} + \nu_s(COO)_{maleate}$
1215	1221	-	Ag	$\nu(CC)_{phen} + \nu(CN)_{phen} + \nu_s(COO)_{maleate}$
1223	-	1226	Au	$\nu(CC)_{phen} + \nu(CN)_{phen} + \nu_s(COO)_{maleate}$
1255	1267	-	Ag	$\nu(CC)_{phen} + \nu(CN)_{phen} + \nu_s(COO)_{maleate}$
1290	1283	-	Ag	$\nu(CC)_{phen} + \nu(CN)_{phen} + \nu_s(COO)_{maleate}$
1315	-	1318	Au	$\nu(CC)_{phen} + \nu(CN)_{phen} + \nu_s(COO)_{maleate}$
1315	1323	-	Ag	$\nu(CC)_{phen} + \nu(CN)_{phen} + \nu_s(COO)_{maleate}$
1331	1334	-	Ag	$\nu(CC)_{phen} + \nu(CN)_{phen} + \nu_s(COO)_{maleate}$
1356	-	1349	Au	$\nu(CC)_{phen} + \nu(CN)_{phen} + \nu_s(COO)_{maleate}$
1407	-	1402	Au	$\nu(CC)_{phen} + \nu(CN)_{phen} + \nu_s(COO)_{maleate}$
1407	1418	-	Ag	$\nu(CC)_{phen} + \nu(CN)_{phen} + \nu_s(COO)_{maleate}$
1436	-	1430	Au	$\nu(CC)_{phen} + \nu(CN)_{phen} + \nu_s(COO)_{maleate}$
1437	1435	-	Ag	$\nu(CC)_{phen} + \nu(CN)_{phen} + \nu_s(COO)_{maleate}$
1482	1465	-	Ag	$\nu(CC)_{phen} + \nu(CN)_{phen} + \nu_s(COO)_{maleate}$
1497	-	1495	Au	$\nu(CC)_{phen} + \nu(CN)_{phen} + \nu_s(COO)_{maleate}$
1497	1502	-	Ag	$\nu(CC)_{phen} + \nu(CN)_{phen} + \nu_s(COO)_{maleate}$
1518	1508	-	Ag	$\nu_{as}(COO)_{maleate} + \nu(CC)_{phen} + \delta(H_2O)$

1517	-	1517	Au	$\nu_{as}(COO)_{maleate} + \nu(CC)_{phen} + \delta(H_2O)$
1527	1528	-	Ag	$\nu_{as}(COO)_{maleate} + \nu(CC)_{phen} + \delta(H_2O)$
1557	-	1563	Au	$\nu_{as}(COO)_{maleate} + \nu(CC)_{phen} + \delta(H_2O)$
1569	-	1572	Au	$\nu_{as}(COO)_{maleate} + \nu(CC)_{phen} + \delta(H_2O)$
1600	1599	-	Ag	$\nu_{as}(COO)_{maleate} + \nu(CC)_{phen} + \delta(H_2O)$
1623	1617	-	Ag	$\nu_{as}(COO)_{maleate} + \nu(CC)_{phen} + \delta(H_2O)$
1622	-	1624	Au	$\nu_{as}(COO)_{maleate} + \nu(CC)_{phen} + \delta(H_2O)$
1635	1637	-	Ag	$\nu_{as}(COO)_{maleate} + \nu(CC)_{phen} + \delta(H_2O)$
1665	-	1653	Au	$\nu_{as}(COO)_{maleate} + \nu(CC)_{phen} + \delta(H_2O)$
1664	1664	-	Ag	$\nu_{as}(COO)_{maleate} + \nu(CC)_{phen} + \delta(H_2O)$
2968	-	2836	Au	$\nu(CH)_{phen}$
2996	-	2901	Au	$\nu(CH)_{phen}$
2964	2961	-	Ag	$\nu(CH)_{phen}$
2995	2994	-	Ag	$\nu(CH)_{phen}$
3067	3048	-	Ag	$\nu(CH)_{phen}$
3066	-	3068	Au	$\nu(CH)_{phen}$
3098	3078	-	Ag	$\nu(CH)_{phen}$
3104	3099	-	Ag	$\nu(CH)_{phen}$
3112	3112	-	Ag	$\nu(CH)_{phen}$
3125	3126	-	Ag	$\nu(CH)_{phen}$
3126	3149	-	Ag	$\nu(CH)_{phen}$
3127	3191	-	Ag	$\nu(CH)_{phen}$
3154	3200	-	Ag	$\nu(CH)_{phen}$
3345	3222	-	Ag	$\nu(OH)_{H_2O}$
3161	-	3223	Au	$\nu(CH)_{phen}$
3347	3278	-	Ag	$\nu(OH)_{H_2O}$
3346	-	3409	Au	$\nu(OH)_{H_2O}$

†Nomenclature: Tr – translation; τ – torsion; δ – bending; Φ – out-of-plane bending; ν – stretching; ν_s – symmetric stretching; ν_{as} – anti-symmetric stretching.
